# Relapsed/Refractory Multiple Myeloma in 2020/2021 and Beyond

**DOI:** 10.3390/cancers13205154

**Published:** 2021-10-14

**Authors:** Klaus Podar, Xavier Leleu

**Affiliations:** 1 Department of Internal Medicine 2, University Hospital Krems, Mitterweg 10, 3500 Krems an der Donau, Austria; 2 Molecular Oncology and Hematology Unit, Karl Landsteiner University of Health Sciences, Dr. Karl-Dorrek-Straße 30, 3500 Krems an der Donau, Austria; 3 Department of Hematology, and CIC1402 INSERM Unit, Poitiers University Hospital, 2 Rue de la Milétrie, 86021 Poitiers, France; xavier.leleu@chu-poitiers.fr

**Keywords:** relapsed/refractory multiple myeloma (RRMM), novel therapies, immunotherapies, CAR T cells, bispecific antibodies, clonal evolution, protein degradation

## Abstract

**Simple Summary:**

During the last two decades, collaborative translational studies utilizing novel methodologies have dramatically advanced our understanding of multiple myeloma (MM) pathophysiology and revolutionized derived treatment strategies. Nevertheless, MM remains an incurable disease, with the vast majority of patients relapsing due to evolving genetic alterations within tumor cell clones as well as the pressure of the immunosuppressive bone marrow (BM) microenvironment. Therefore, continuous translational efforts are needed to further increase our understanding of MM biology, develop rationally derived drugs and to thereby improve patient outcome.

**Abstract:**

Despite the challenges imposed by the COVID-19 pandemic, exciting therapeutic progress continues to be made in MM. New drug approvals for relapsed/refractory (RR)MM in 2020/2021 include the second CD38 monoclonal antibody, isatuximab, the first BCMA-targeting therapy and first-in-class antibody–drug conjugate (ADC) belantamab mafodotin, the first BCMA-targeting CAR T cell product Idecabtagen-Vicleucel (bb2121, Ide-Cel), the first in-class XPO-1 inhibitor selinexor, as well as the first-in-class anti-tumor peptide-drug conjugate, melflufen. The present introductory article of the Special Issue on “Advances in the Treatment of Relapsed and Refractory Multiple Myeloma: Novel Agents, Immunotherapies and Beyond” summarizes the most recent registration trials and emerging immunotherapies in RRMM, gives an overview on latest insights on MM genomics and on tumor-induced changes within the MM microenvironment, and presents some of the most promising rationally derived future therapeutic strategies.

## 1. Introduction

Multiple myeloma (MM), defined as a malignant disorder of post-germinal center (GC) B cells, is characterized by the clonal proliferation of plasma cells (PCs) leading to hypercalcemia, renal insufficiency, anemia, and bone disease (CRAB criteria) or bone marrow (BM) infiltration with clonal PCs > 60%, serum FLC ratio > 1:100, and/or more than one lesion on MRI or PET/CT (Myeloma-Defining Events or SLiM CRAB criteria), as well as immunodeficiency [1]. With the introduction and continuous improvement of molecular, genetic and cellular technologies, collaborative preclinical studies have dramatically advanced our understanding of genetic and molecular MM pathophysiology during the past two decades. Functionally, we now know that accumulating genomic alterations within tumor cells as well as MM-specific vulnerabilities (i.e., the high dependency of tumor cells on the ubiquitin-proteasome system [UPS]) and MM-induced changes within the bone marrow (BM) microenvironment, result in tumor cell proliferation, survival, migration, and drug resistance as well as osteolysis, BM angiogenesis, and immunosuppression. These findings led to the approval of 15 novel agents and a total of 33 novel regimens for MM therapy during the last two decades. The bench-to-bedside translation of Immunomodulatory drugs (IMiDs; lenalidomide, pomalidomide), proteasome inhibitors (PIs; bortezomib, carfilzomib, ixazomib), and monoclonal antibodies (mAbs; elotuzumab, daratumumab, isatuximab), in particular, has revolutionized MM therapy and resulted in the improvement of patient survival from 3–4 years in the 1990s to currently more than eight years. 

**Figure 1:**
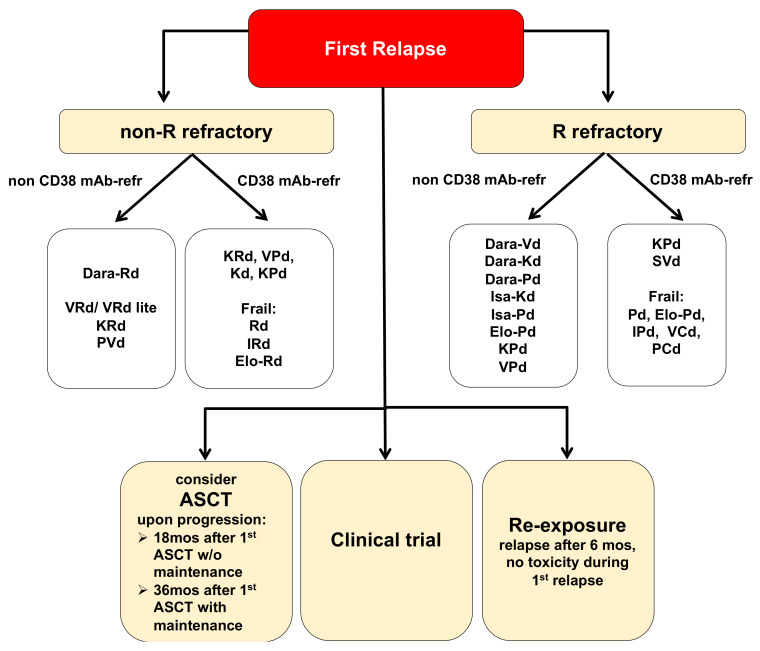
Treatment options for multiple myeloma (MM) patients at first relapse. Listed treatment options are selected and not inclusive of all available regimens. Evidence for these treatment regimens stems from randomized-controlled trials discussed in this review article. Dara, daratumumab; K, carfilzomib; R, Revlimid/lenalidomide; P, pomalidomide; V, Velcade/bortezomib; I, ixazomib; Elo, elotuzumab; Isa, isatuximab; C, cyclophosphamide; ASCT, autologous stem cell transplantation.

Nevertheless, relapse is still inevitable in the majority of patients, including those who achieved deep remissions becoming progressively shorter [2]. Criteria for relapsed/refractory multiple myeloma (RRMM) include the IMWG criteria for progressive disease (PD), progressive disease on treatment or after at least minimal response (MR), or progressive disease ≤60 days following the most recent treatment, the absence of at least MR on a given therapy (primary refractory disease), the presence of PD criteria in the absence of features for RRMM, or primary refractory MM [1,2,3,4]. A note of caution: given that continuous treatment has become standard, most RRMM patients need to be considered treatment-refractory. A revision of current IMWG criteria for RRMM should therefore be considered. While exclusive monitoring may be considered for MM patients with asymptomatic biochemical relapse with slow tempo, immediate treatment is required for those with cytogenetic high-risk features, with renal or neurologic complications, and with a rapid doubling of the M-spike. Therapy-related features (response to/toxicity of/exposure/refractoriness to prior therapies, SCT eligibility, prior SCT), disease-related features (genetic alterations, duration of prior remission, extramedullary disease, high tumor burden, pace of disease, end-organ function), and patient-related features (performance status, comorbidities, disabilities, patient preference, accessibility to treatment centers, compliance, cost) guide the choice of treatment in RRMM patients. Treatment goals vary among patients with RRMM and should consider disease control, extension of survival, and maintenance of quality of life (QoL). Specifically, in frail MM patients, treatment should focus on symptom relief rather than on attaining a deep and durable response. In contrast, in patients with aggressive disease, treatment should be initiated immediately. **Current treatment strategies in RRMM** [3,4] include a change of therapy regimens (relapse after < 6 months), a re-challenge with previous therapy regimens (relapse after > 6 months), autologous SCT (progression after > 18 months for patients who are not on maintenance, and after > 36 months for patients who are on maintenance), or inclusion into a clinical trial (Figure 1). Triplet regimens should always be preferred. 

Besides briefly summarizing updates on clinical trials that led to regulatory drug approvals in RRMM during the last months, the present introductory article will give an overview of the most recent advances of our knowledge on RRMM biology, MM genomics and their evolving impact on personalized treatment strategies. Moreover, we will introduce the reader to venetoclax, CELMoDs, additional CAR T cell products, and bispecific antibodies, but also to *proteolysis targeting chimeras* (PROTACs)/degronomids that ensure the continuing rapid treatment progress in (RR)MM.

Eminent experts will then discuss in comprehensive state-of-the-art articles the therapeutic developments in RRMM and offer evidence-based recommendations for combinatorial treatment approaches. Topics include up-to-date diagnosis and treatment response monitoring in RRMM, risk-adapted stratification and sequencing of RRMM therapies, therapeutic advances in RRMM patients with renal insufficiency, novel non-immunologic agents for RRMM patients, and the use of bispecific antibodies in RRMM, and the management of adverse events and supportive therapy in RRMM. 

## 2. Recent Registration Trials in the Context of Current Treatment Standards

### 2.1. Treatment Options for MM Patients in First Relapse

Results of more than 9 phase III trials shape the current treatment landscape **of triplet combination therapies** in RRMM. Revlimid (lenalidomide)/dexamethasone (**Rd)** represents the backbone of 4 of these triplet combinations (KRd, IRd, DRd, Elo-Rd). The addition of the third substance led to an improvement in the depth of the response and subsequently to an improved PFS. A recent follow-up of the POLLUX trial (NCT02076009) demonstrated particularly impressive data with Dara-Rd: ORR 93% (CR 56%), MRD negativity 30% and the longest proven PFS (44.5 months) and PFS2 (NR vs. 31 months) to date. A clear survival advantage is expected. These data support the preferred use of Dara-Rd in the first relapse. However, the unsatisfactory effect of Dara-Rd in the subpopulation of cytogenetically high-risk patients is a caveat [5]. Combination strategies containing PIs (i.e., VRd and VRd lite) may be preferable in this patient population. In view of the extensive use of lenalidomide in the upfront and maintenance setting, the burning question remains, how to best treat **lenalidomide-refractory** patients. Importantly, neither IMiDs nor the PIs are “class-refractory”, meaning co-resistant with other members of these drug classes. Lenalidomide-free therapy options in the first relapse include carfilzomib and dexamethasone (Kd) and daratumumab (Dara)-Vd, and based on the recently published **CANDOR trial** (NCT03158688; Dara-K56d vs. K56d) also Dara-Kd. Dara-K56d showed a 37% reduction in the risk of progression or death in a population that also included lenalidomide and PI-refractory patients. These excellent data led to the approval of this combination in RRMM after 1 to 3 previous therapies on 20 August 2020 (FDA) and 18 December 2020 (EMA), respectively [6]. Of note, efficacy and safety of Dara-dexamethasone with weekly carfilzomib (70 mg/m^2^) is comparable to twice-weekly KdD56 while being more convenient [7]. The use of **pomalidomide-containing triplets** represents another potential treatment approach for lenalidomide-refractory patients. Following V-Pd (**OPTIMISMM trial**, NCT01734928) [8,9] and Elo-Pd (ELOQUENT-3 trial, NCT02654132) [10], drug combinations of isatuximab (ISA, ICARIA trial/NCT02990338) [11] and Dara (EQULEUUS/MMY1001 trial/NCT01998971-and APOLLO trial/NCT03180736) [12] with Pd received drug approval in 2020 and 2021, respectively. Specifically, the **ICARIA trial** is the first phase III study based on which a CD38 mAb in combination with a Pd backbone received approval on March 2020 (FDA) and 22 June 2020 (EMA) for RRMM patients with at least two previous therapies (mPFS 12.7 vs. 7, 9 months; MRD negativity 5.2 vs. 0; significant improvement in PFS2, and a strong trend of improved OS) [13,14]. Data of the phase III **APOLLO trial** (Dara-Pd vs. Pd) are similarly exciting, with a 12-month PFS rate of 52% vs. 35% (HR = 0.63) [15]. A deep response with a clear improvement in PFS for Isa-K56d (NR vs. 19 months, very good partial remission [VGPR] 72.6% vs. 56.1%, MRD negativity 29.6% vs. 13%) was also observed in the phase III **IKEMA trial** (NCT03275285, Isa-Kd vs. Kd) [16]. Based on these data Isa-Kd received approval for the treatment of adult patients with RRMM who have received one to three prior lines of therapy on 31 March 2021 (FDA) and 19 April 2021 (EMA), respectively. Of note, a PFS benefit was also seen in patients with HR cytogenetics del17p, t(4;14), t(14;16), 1q21 gain; as well as in patients older than 70 years; regardless of the refractory status or the number of prior treatment lines [17,18,19]. 

In summary, for the treatment of RRMM triplets with second generation IMiDs, PIs, and monoclonal antibodies are preferred upon relapse. Dara-Rd, Dara-Vd, Dara-Kd are recommended as second-line options, with Dara-Vd and Dara-Kd being also active in lenalidomide-resistant relapse. Elo-Rd is recommended as second-line therapy for patients with lenalidomide-sensitive disease, and may also be considered after ≥3 prior lines of therapy in combination with Pd for refractory disease. Third line treatment options are predominantly guided by drug-refractoriness and frailty. However, while switching therapies (even within the same class) is certainly a commendable strategy, drug refractoriness may also be overcome by the addition of an additional agent. A clear recommendation for second-line therapy of Dara-pre-treated (Dara-refractory) patients is currently still pending. Dependent on the backbone used during previous therapy, the use of KRd, IRd, Elo-Rd, Rd, but also PVd, Kd, and KPd are possible. Available data on whether isatuximab is effective in Dara-refractory patients or vice versa currently do not support the switch of CD38-targeting agents (Table 1, Figure 1). 

### 2.2. Treatment Options for MM Patients in Second or Higher Relapse 

The incorporation of lenalidomide, pomalidomide, bortezomib and second-generation proteasome inhibitors as well as of monoclonal antibodies in the upfront setting as well as at first relapse results in heavily pre-treated populations at second or higher relapses. Triple (1 IMiD, 1 PI, 1 anti-CD38)-, quad (2 IMiDs, 1 PI, 1 anti-CD38 or 1 IMiD, 2 PIs, 1 anti-CD38)-or penta (2 IMiDs, 2 PIs, 1 anti-CD38)-refractory patients become increasingly frequent. Based on the retrospective MAMMOTH study (*n* = 275) median OS in MM patients (1) not triple refractory was 11.2 months; (2) triple/quad-refractory (*n* = 148) was 9.2 months; and (3) penta-refractory (*n* = 70) was 5.6 months [20]. 

Selinexor is an inhibitor of the nuclear export protein XPO-1, which blocks the nuclear export of tumor suppressors, the glucocorticoid receptor and eIF4E-bound mRNAs (c-Myc, Bcl-XL, MDM2) [21]. Based on data from the **STORM trial** (NCT02336815; ORR 26%, median PFS 4 months, median OS 9 months), selinexor 80 mg twice weekly in combination with dexamethasone was approved on 3 July 2019 (FDA) and on 29 March 2021 (EMA), respectively, for the treatment of penta-refractory RRMM patients with ≥4 previous therapies [22]. Even if the toxicity of this therapy (thrombopenia, fatigue, GI toxicity) has been widely discussed, results obtained in this heavily pre-treated patient population are convincing. Ongoing trials are examining selinexor in combination with other substances in earlier lines of therapy. Final data from the **BOSTON trial** (NCT03110562; S-Vd vs. Vd) demonstrated a significantly improved response rate (ORR 76.4 vs. 62.3%; mPFS 13.93 vs. 9.46 months) and reduced toxicity for S-Vd vs. Vd in a weekly selinexor-dosing strategy [23]. Based on these data S-VD has been approved for the treatment of RRMM patients who have received at least 1 prior therapy on 21 December 2020 (FDA). Early data of the ongoing **STOMP trial** (NCT02343042), which investigates selinexor in combination with different standard-of-care agents including weekly-dosing of selinexor in combination with Kd, Pd, and Dara are also promising [24,25,26,27]. A direct comparison with anti-CD38-containing combinations such as Dara-Pd or Isa-Pd is not possible. Nevertheless, selinexor may represent an excellent option for all oral triplet therapies. Moreover, selinexor-based triplets may be considered as a strategy to bridge to CAR T cell therapy or for RRMM patients who are not eligible to CAR T cell-, BiTE-, or ADC-therapy. Phase III trials which compare different triplets would be necessary to determine whether selinexor-containing triplets may be preferred, at least in subgroups of RRMM patients.

**Figure 2:**
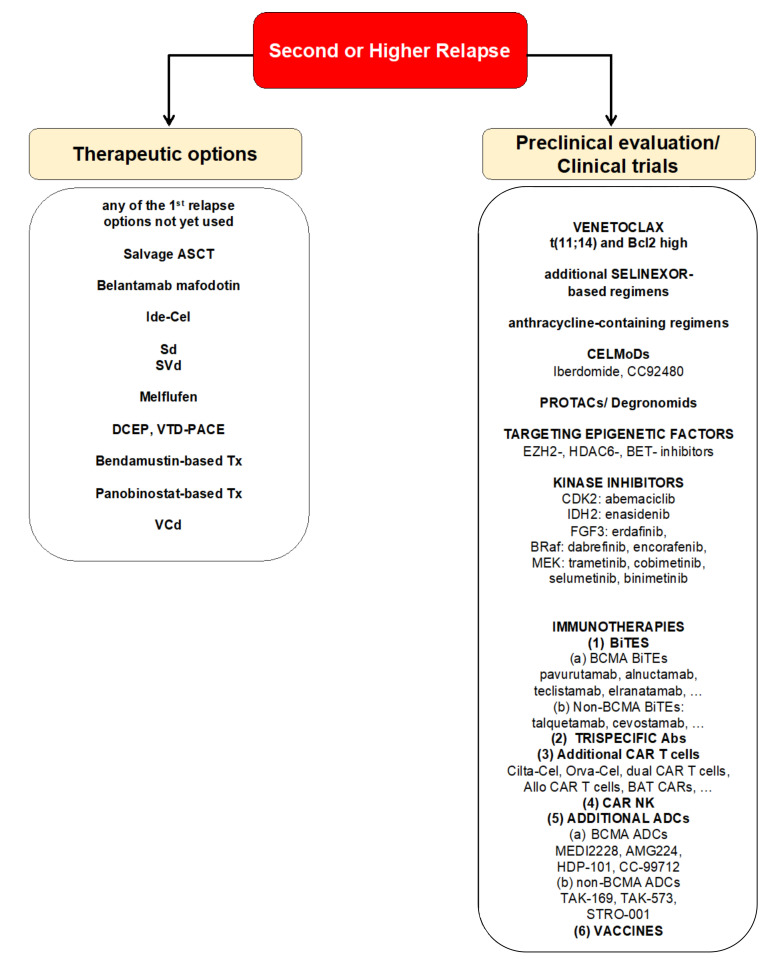
Treatment options for MM patients with a second or higher relapse. Listed treatment options are selected and not inclusive of all available regimens. Evidence for these treatment regimens stems from randomized-controlled trials discussed in this review article. S, Selinexor; PROTACs, proteolysis-targeting chimera; BCMA, B cell maturation antigen; DCEP, dexamethasone/cyclophosphamide/etoposide/cisplatin; VTD-PACE, V (bortezomib), T (thalidomide), D (dexamethasone), PACE (cisplatin/doxorubicin/cyclophosphamide/etoposide).

The lipophilic alkylant melflufen is the first anti-tumor peptide-drug conjugate. Due to its lipophilicity, it is easily taken up into tumor cells and cleaved to the active alkylane by aminopeptides, which are present in increased numbers in tumor cells. Upon release, melphalan alkylates the DNA and induces tumor cell apoptosis. In continuation of the O-12-M1 trial (NCT01897714) promising results were obtained in the phase II **HORIZON trial** (NCT02963493) which investigated the anti-MM activity of melflufen in combination with dexamethasone in heavily pre-treated (26% triple-refractory) MM patients (ORR of 31%, a PFS of 5.7 months, and an mOS of 20 months) [28]. Based on these data melflufen in combination with dexamethasone was approved for patients with RRMM who have received at least 4 prior therapies and whose disease is refractory to at least one IMiD, one PI, and one CD38-directed mAb on 26 February 2021. We propose that optimal candidates for this regimen are patients with alkylator-sensitive disease with good baseline counts (ANC ≥ 1.0, plts ≥ 100). First data of the phase III **OCEAN trial** (NCT03151811) with melflufen/dexamethasone vs. pomalidomide/dexamethasone are expected later this year. Melflufen could be an excellent option for RRMM patients with alkylator-sensitive disease and good baseline blood counts. However, it needs to be mentioned that a partial hold was recently put on all trials with melflufen due to an increased risk of death associated with melflufen/dexamethasone vs. pomalidomide/dexamethasone in RRMM patients after 2–4 prior lines of therapy (Table 1, Figure 2).

Besides novel treatment regimens, salvage ASCT remains another treatment option to be considered. Its use varies widely. Recent retrospective data of the *Center for International Blood and Marrow Transplant Research* (CIBMTR) strongly indicated a role of salvage ASCT in the novel agent era with a 1-year PFS of 50% and an OS of 94%. It thereby compares favorably with several other approved regimens using newer agents [29]. Another retrospective study performed by the *European Blood and Marrow Transplant group* (EBMT), showed a median OS of 7 months after the third ASCT if the relapse-free interval (RFI) was <six months, 13 months if the RFI was between 6 and 18 months, and 27 months if the RFI was ≥18 months [30]. The only prospective phase III trial demonstrated that salvage ASCT transplant conferred the highest benefit when performed as consolidation after the second line chemotherapy rather than later in the treatment course [31]. In summary, although effective, modern randomized studies are required to better define the role of salvage ASCT as part of evolving treatment strategies in RRMM.

### 2.3. Evolution of Immunotherapies for RRMM Patients

While **naked** mAbs directed against SLAMF7 (elotuzumab) and CD38 (Dara, ISA) have become therapeutic backbones in RRMM, **allogeneic stem cell transplantation** (ORR 80%, median PFS 20 months) and **donor lymphocyte infusions**, which mediate a donor T-lymphocyte-mediated graft-versus-myeloma effect, remain restricted to few patients due to its high toxicity and mortality rate. With the establishment of various new immunotherapeutic approaches, such as Antibody–Drug Conjugates (**ADCs**), Bispecific T-Cell Engagers (**BiTEs**) and Chimeric-Antigen-Receptor (**CAR**) T cells, treatment regimens in RRMM are currently revolutionizing MM therapy.

#### 2.3.1. Antibody Drug Conjugates (ADCs)

B-cell maturation antigen (“B-cell maturation antigen”, BCMA) represents the predominant point of attack of these modalities [32,33]. The **ADC** belantamab mafodotin (Belamaf, Blenrep) is a mAb that is linked to the cytotoxin monomethyl-auristatin F (MMAF) and induces MM cell death via antibody-dependent cytotoxicity (ADC), antibody-dependent cell-mediated cytotoxicity (ADCC), immunogenic cell death (ICD) and direct BCMA receptor inhibition. Based on the **DREAMM-2 trial**, belantamab mafodotin became the first-in-class ADC approved for monotherapy in patients with RRMM, after ≥ 4 prior therapies, who are refractory to at least one PI, one IMiD and one CD38 mAb on 2 August 2020 (FDA) and 26 August 2020, respectively. Of note, response time in this heavily pre-treated patient population was more than 13 months. Keratopathies occurred in about 50% of patients but did not lead to blindness and were reversible [34]. Nevertheless, belantamb mafodotin should be avoided in patients with preexisting ocular disease. Several ongoing trials investigate belantamab mafodotin in a reduced dose in combination with Pd, pembrolizumab, Rd, Vd, and VRd, and also in earlier lines of therapy (DREAMM-3 to -9). Moreover, DREAMM-12 and -13 evaluate belantamab mafodotin safety in RRMM patients with renal or hepatic impairment, respectively. Other ADCs in early clinical evaluation include BCMA-targeted ADCs MEDI2228, AMG224, HDP-101 and CC-99712, CD38-targeted ADCs TAK-169 and TAK-573, and the CD74-targeted ADC STRO-001 [35,36,37,38,39,40] (Table 1, Figure 2).

#### 2.3.2. Bispecific Antibodies (BiTEs/T Cell Engagers)

The biggest excitement during the last months came with the clinical introduction of a new class of therapeutic agents in MM, the *Bispecific T cell Engagers* (**BiTEs**). They link a surface target molecule (i.e., BCMA) on tumor cells to CD3 on T cells and thereby redirect activated T cells to induce tumor cell death. The first-in-human BiTE directed against BCMA, **AMG420 (BI-836909)**, showed an ORR of 70% and a response duration of 5.6 to 10.4 months in RRMM patients. However, a 4-weeks on and 2 weeks off continuous i.v.-treatment protocol for 6-week cycles posed a significant challenge, why the further development of AMG420 has been suspended [42]. Preliminary results of a trial investigating the half-life-extended anti-BCMA BiTE **AMG701 (pavurutamab)** are encouraging (ORR 83%) [43]. Excitingly, **CC-93269/alnuctamab**, a humanized 2 + 1 BCMA/T cell engager, resulted in ORR of up to 89% and a sCR of 44% in heavily pre-treated patients (66.7% triple refractory) [44,45]. The phase I **MajesTEC-1** trial (NCT04557098) on the efficacy and tolerance of the subcutaneous “off-the-shelf” BCMA/CD3-BiTE **teclistamab** (JNJ-64007957) demonstrated an ORR 65% (>58% VGPR, >40% CR) in an even heavier pre-treated patient population (triple-class refractory 80%; penta-refractory 41%). The drug was well tolerated with no high-grade *Cytokine Release Storm* (CRS), and self-limited cytopenias. Given confirmatory results in the phase II extension of this trial teclistamab is likely to become the first bispecific antibody approved for the therapy of RRMM [46]. Further trials examining teclistamab in earlier lines of therapy are planned. Other BCMA/CD3-BiTEs currently under clinical evaluation include REGN5458 and TNB-383B [47,48]. Very early results of the **MagnetisMM-1 trial** (NCT03269136) on the use of the subcutaneously administered, humanized BCMA-CD3 bispecific antibody **elranatamab** (PF-06863135) in patients with a median of 8 prior lines of therapy were comparable to those with teclistamab, and also included patients previously treated with BCMA-targeting agents [49]. However, it needs to be noted that the trial was transiently on hold due to the development of neuropathy in some patients. While clinical trials predominantly focus on BCMA/CD3-BiTEs, there is an urgent need for alternatively targeting BiTEs. **Non-BCMA-targeting BiTEs** are already under early clinical phase I evaluation in RRMM; they include the subcutaneous GPRCD5/CD3-BiTE **talquetamab** (JNJ-64007957) (NCT03399799/MonumentTAL-1, NCT04634552 part 1-2/MonumentaTAL-1 part 3, NCT04108195/TRIMM-2/MMY1002, NCT04586426/RedirecTT-1) and the FcRH5/CD3-BiTe **cevostamab** (BFCR4350A) (NCT03275103). Early data on talquetamab and cevostamab suggested a manageable safety profile with high, rapid and durable response rates (ORR ~60–70%) that deepened over time in a group of heavily pre-treated patients, who have also received BCMA-targeted therapy [50,51]. An additional study is ongoing with the CD38/CD3 BiTEs GBR1342 (NCT0330911); studies with trispecific CD38/CD28/CD3 antibodies are planned. Moreover, based on preclinical observations, clinical trials investigating teclistamab in combination with other anti-MM agents (for example, IMiDs or daratumumab) (NCT04722146, NCT04108195/TRIMM-2/MMY1002) and/or talquetamab (NCT04586426) in RRMM are currently recruiting. Another option of BiTE-containing combination therapy may be a vaccination approach, and recent data demonstrated that a PLGA/heteroclitic BCMA72-80 peptide induces HLA-A2 restricted central and effector memory cytotoxic T cells (CTLs) and thereby increased granzyme-mediated cytotoxicity of BCMA-BiTEs [52]. The development of resistance against monoclonal antibodies represents a key challenge in the treatment of MM. Mechanisms of resistance include low expression of the surface target gene due to selection of cells with low target expression; transfer of the tumor antigen to T cells followed by BiTE-induced fratricide of T cells (trogocytosis); overexpression of the complement-inhibitory proteins CD55 and CD59; depletion of NK cells (by CD38 antibodies); decreased activation of T cells and macrophages; as well as deregulation of regulatory T cells, tumor-associated macrophages, and myeloid-derived suppressor cells. Being available “off-the-shelf”, BiTEs but also ADCs represent community practice-friendly choices, for patients with rapid disease progression in particular. Nevertheless, in contrast to CAR T cells, an intensive treatment schedule is required for these modalities, thereby challenging the patient’s QoL [34,46].

#### 2.3.3. Chimeric Antigen Receptor T Cells (CAR T Cells)

Investigations on the therapeutic use of **CAR-T cells** remains the “hot topic” in RRMM also in 2020/2021; several excellent and comprehensive reviews have been published recently (e.g., [53]). Excitingly, CAR T cell treatment is a one-time treatment with the highest efficacy among the novel BCMA-targeting immunotherapeutic options [54]. Confirming previous data of a phase I trial [55], a 25 month-update of the pivotal **phase II KarMMa** trial (NCT03361748) on the anti-MM activity of the BCMA-CAR T cell product **Idecabtagen-Vicleucel (bb2121, Ide-Cel)** demonstrated an ORR of up to 82% (sCR/CR 33%) and a median PFS of 12.1 months (20.2 mos in patients with CR or sCR) at 450 × 10^6^ CAR T cells in a heavily pre-treated patient population (median six prior therapies; 84% triple refractory). Of note, responses were independent of age or risk, and included patients with extramedullary, triple-or penta-refractory disease [41]. Based on these data, Ide-Cel was approved as the first BCMA-directed CAR T cell product for the treatment of RRMM patients after ≥4 prior lines of therapy, including an immunomodulatory agent, a proteasome inhibitor, and a CD38 monoclonal antibody on 27 March 2021 (FDA) and 19 August 2021 (EMA), respectively. In order to enrich CAR T cells with a memory-like phenotype, the bb2121 CAR T cell product was treated ex vivo with the PI3-kinase inhibitor bb007, therefore called bb21217 [56,57]. Exciting interim results were presented this year also on another BCMA-CAR-T cell product, **Ciltacabtagen-Autoleucel (Cilta-Cel, JNJ-68284528 or JNJ-4528 or LCAR-B38M)**, which consists of a tandem antigen receptor for BCMA. After a follow-up of 18 months, the **phase Ib/II CARTITUDE-1** trial (NCT03548207) reported an impressive ORR 97.9%, with a sCR 80.4% and an OS 81% in MM patients with an average of 6 prior therapies. Of note, 66% of all patients were still in remission after 18 months; this value was even higher in those patients who had achieved a sCR (75.9%). It is noteworthy that of 61 patients with evaluable MRD status, 91.8% were MRD negative (cut-off: 10^−5^). Cilta-Cel has been granted breakthrough therapy designation status by the US FDA in early 2021. The regulatory approval of Cilta-Cel both in the USA and the EU is expected within the next months. Several clinical trials examining the anti-MM activity of Cilta-Cel and Ide-Cel in earlier stages of the disease are ongoing [58]. Promising initial results of the **phase II CARTITUDE-2** trial (NCT04133636) in RRMM patients after one to three prior lines of therapy were presented at this year’s ASCO meeting (after a six months follow-up: ORR 95%, ≥CR 75%) [59]. Data on Ide-Cel and Cilta-Cel suggest that Ide-Cel may be more favorable in terms of tolerability, while Cilta-Cel is slightly more impressive in terms of efficacy. Exciting early results have also been presented for **Orvacabtagen-Autoleucel (Orva-Cel, JCARH125)**, a fully human BCMA-CAR T cell product characterized by a modified spacer and a defined CD4: CD8 ratio. The multicenter phase I/II **EVOLVE trial (NCT03430011)** in patients with RRMM after at least 3 prior lines of therapy (94% triple-refractory, 48% penta-refractory) demonstrated an ORR 92% (sCR/CR 36%), with 69% of patients maintaining CAR T cell persistence after 6 months [60]. CRS rates were 60 to 80% for BiTes and 60 to 80% for CAR T-cell products; Immune Effector Cell-associated Neurotoxicity Syndrome (ICANS) rates were 10 to 20% for CAR T cells. CAR T cells should therefore be avoided in frail patients with significant cardiopulmonary or neurologic disorders who are likely to not tolerate CRS or ICANS; or in patients with a rapid relapse in need of immediate therapy. Despite unique response rates (i.e., high MRD negativity) even in heavily pre-treated MM patients, the durability of CAR T cells needs to be further improved. PFS lasts a maximum of one year without developing a plateau, indicative for a loss of response over time and disease relapse. Ongoing trials therefore investigate whether the use of CAR-T cells in earlier lines of therapy or combination strategies eradicate the malignant clone, prolong the response time and improve tolerance. 

Moreover, we need to better understand mechanisms of resistance against CAR T cell therapy; contributing factors include CAR T cell-and tumor cell-intrinsic features (i.e., poor T cell expansion and persistence; impaired T cell function via exhaustion resistance, high tumor burden or tonic signaling; tumor cell heterogeneity with changes in target antigen expression via clonal selective pressure, trogocytosis, splice variants, and lineage switch), features mediated via the microenvironment (e.g., immune suppression), as well as impaired T cell trafficking [61,62]. Specifically, recently discovered mechanisms-of-actions include biallelic BCMA-loss [63,64] or the inhibitory effect of T regs [65], which induce resistance against BCMA-targeting agents [66]. Counteracting therapeutic strategies to overcome resistance against BCMA-targeting CAR T cells may include the use of non-BCMA CD38-, SLAMF7-, GPRC5D-, CD138-, ikappa light chain-targeting CAR T cells; as well as dual-targeted, triple CAR T cells such as BCMA/CD38/CD3-or CD19/BCMA/CD3-CAR T cells [67,68,69]; or also off-the-shelf AlloCAR T cells. Very early results of the UNIVERSAL trial (NCT04093596), a first-in-class human study with the AlloCAR T cell product ALLO-715 CAR T + ALLO-647 anti-CD52 mAb are promising (ORR 60%, ≥VGPR 40%; no GvHD) [70]. Additional strategies under investigation include CAR T cell-containing-combination therapies with IMiD/CELMoD agents, anti-CD38 monoclonal antibodies, checkpoint inhibitors, and the use of gamma secretase inhibitors. Besides CAR T cells, CAR-NK cells against BCMA (e.g., FT576, NK92, UCB), CD38, SLAMF7 and NKG2D are under development with the potential advantage of multiple off-the-shelf sources, lower toxicity and mode-of-actions via CAR but also endogenous receptors [71,72]. Clinical trials have already been initiated (e.g., NCT03940833, NCT05008536) [73].

Moreover, several ongoing preclinical strategies also seek to improve the clinical practicability of CAR T cells and BiTEs. For example, recent data suggest the therapeutic potential of transiently active anti-BCMA mRNA-versus DNA-based CAR T cells. These CAR T cells could be given in regular intervals, thereby increasing anti-MM efficacy while reducing toxicity [74]. As another exciting option, the use of *Binary Activated T Cells with Chimeric Antigen Receptors* (BAT-CARs) directed against “molecule X”-labelled antibodies comes with the promise of creating a wider therapeutic window, of expanding the use of existing targets (i.e., already available multiple antibodies can be labelled with the same molecule), and of preventing antigen escape and therefore the development of resistance (Table 1, Figure 2). 

## 3. The Therapeutic Future of RRMM

**Functionally**, the development of RRMM is based on changes of the **intrinsic tumor cell biology** (e.g., evolving genomic sub/clonal aberrations; evolving mechanisms of resistance against initial therapies; deregulated signaling pathways; deregulated apoptotic and autophagic programs), of **the tumor microenvironment** (e.g., evolving deregulation of the finely tuned homeostasis within the bone marrow, resulting in an increased immunosuppression in particular), but also of **host factors** (e.g., treatment intolerance, organ failure, frailty). Ongoing efforts aim to improve the use and sequencing of, and to identify resistance mechanisms against existing treatment regimens, but also to discover novel therapeutic targets, to develop rationally derived agents and to integrate them into forthcoming treatment strategies. For an excellent up-to-date summary of our knowledge on mechanisms mediating resistance against IMiDs, PIs, CD38 mAbs, and BCMA-targeting agents as well as emerging therapeutic strategies to overcome them, we refer to a comprehensive recent review article by Drs Davis et al. [75]. Of interest, a very recent study demonstrated that resistance against CD38-targeting mAbs is, at least in part, related to microenvironment-mediated downregulation of CD38 on tumor cells. Indeed, JAK2 inhibitor ruxolitinib inhibits Stat3-dependent, bone marrow stroma cell (BMSC)/IL-6-mediated downregulation of CD38 and thereby restores sensitivity to anti-CD38 agents, and anti-CD38-triggered ADCC, in particular [76]. Our current knowledge on evolving molecular and generic alterations in tumor cells and cells of the BM microenvironment as well as on derived novel approaches of treatment stratification and personalization in RRMM will be summarized below. Adverse events mediated by these agents will be comprehensively summarized by Drs Pozzi and colleagues in this special issue. 

### 3.1. Molecularly-Based Innovative Approaches of Treatment Stratification and Personalization 

#### 3.1.1. Recent Insights into MM Genomics and the Impact of the MM Microenvironment in RRMM

Molecular events contributing to RRMM include: (1) chromosomal translocations, gains and deletions, which are determined by cytogenetic approaches; and (2) driver/point mutations, epigenetic aberrations, and increased genomic instability, which are determined by genomic approaches. 

*Targeting cytogenetic events.***Early cytogenetic studies (FISH analysis)** have defined three stages of myelomagenesis: (1) initial events leading to the transformation of normal plasma cells to MM precursor stages; (2) late events leading to the progression of MM precursor stages to MM; (3) events leading to extramedullary disease and plasma cell leukemia. **Based on refined technologies** (whole genome sequencing, WGS; whole exome sequencing, WES; comparative genomic hybridization, CGH; copy number array, CAN; spectral karyotyping, multiplex FISH analysis), recent studies demonstrated that MM progression is based on the molecular and clonal behavior of tumor cells, and occurs either through **branching evolution** of reservoir tumor cell clones (Darwin principle, in 80% of RRMM) early in myelomagenesis via interaction of tumor cells with the tumor microenvironment; or through **neutral evolution** (in 20% of RRMM), independent of the tumor microenvironment, via the oncogenic impact of driver mutations (i.e., high-risk 14q translocations). Of note, the branching evolution pattern has been associated with inferior overall survival (HR = 2.61, *p* = 0.0048) [77]. **Priming “genetic hits”** in germinal center (GC) B cells trigger signaling pathways that induce a clonal advantage to plasma cells and thereby lead to the evolution of MM precursor stages MGUS and SMM. Defined by early cytogenetic studies, these disease-initiating events include **hyperdiploidy** (~45%) with multiple trisomies (i.e., gain of odd numbered chromosomes 3,5,7,9,11,15,19, and 21) or **non-hyperdiploidy/hypodiploidy** with a high incidence of recurrent chromosomal translocations (~55%). Generated by aberrant class switch recombination (CSR), chromosomal translocations bring oncogenes under the control of the immunoglobulin H (IgH) switch region 14q32. These oncogenes include CCND1 (t[11;14]), CCND3 (t[6;14]), c-Maf (t[14;16]), FGFR3/MMSET (t[4;14]), MafA (t[8;14]) and MafB (t[14;20]). Together with del[13q], these genetic events have been identified in MM as well as in its precursor conditions MGUS and SMM [78,79,80,81]. In contrast to hypodiploid MM, hyperdiploid MM is associated with lengthened survival. High-risk abnormalities include t[4;14], t[14;16], t[14;20], del17p and gain 1q; double hit MM is defined by two of these abnormalities, and triple hit, when three of these abnormalities are present [82]. **Additional genetic hits** together with the selective pressure of the immunosuppressive BM microenvironment mediate the transition of MM precursor stages to MM, MM relapses, and PCL. During the last few years our understanding of MM genomics has been significantly advanced by utilizing modern technologies. Secondary genetic hits include del17; del1p with loss of CDKN2C, FAF1, and FAM46C; 1q gains with amplification of i.e., CKS1B, ANP32E, Bcl9, and PDZK1; copy-number variations (CNVs); as somatic mutations within the MAPK-, NFkB-pathway and the DNA-repair pathway. Utilizing WGS, today’s most comprehensive genomic analysis tool, additional de novo mutations (e.g., FAM46C, TRAF2, NF1, and XBP1), de novo translocations (MAP3K14), pre-existing (e.g., Tet2) and non-coding mutations (Xbp-1, SCML1, and RBX1) have been identified at MM relapse [83]. Moreover, the existence of oncogenic dependencies between primary translocations and hyperdiploidy, and mutated driver genes and common regions of CNVs recently proposed that primary events pre-determine the genomic landscape of MM and give thereby rise to a subsequent non-random accumulation of genetic hits. For example, t[4;14] is associated with mutations in FGFR3, DIS3, and PRKD2; t[11;14] with mutations in CCND1 and IRF4; t[14;16] with mutations in MAF, BRAF, DIS3, and ATM; and hyperdiploidy and gain 11q with mutations in FAM46C; and MYC rearrangements). Profiles of CNVs acquired at relapse differ substantially among MM subtypes, with hyperdiploid (HRD) tumors evolving predominantly in branching pattern vs. linear pattern in t(4;14) vs. stable pattern in t(11;14). CNA acquisition also differs between subtypes based on CCND expression, with a marked enrichment of acquired del(17p) in CCND2 vs. CCND1 tumors [84]. Importantly, epigenetic aberrations including DNA methylation, histone modification, non-coding RNA and super-enhancers are increasingly recognized to play a key role in MM pathogenesis, the clonal heterogeneity and plasticity of MM in its microenvironment, in particular [85,86]. Most recently, primary molecular events involving SMARC2, NSD2, and PTP4A3 have been identified to affect key epigenetic enzymes, i.e., MMSET [87]. Moreover, super-enhancer profiling has revealed previously unrecognized novel oncogenes MAGI2 and HJURP [88]. Based on the presence of molecular targets **“personalized” therapy concepts** are emerging for MM therapy. Most prominently, **venetoclax**, a highly potent, selective BCL2 inhibitor is active in MM cells with t(11;14) or high Bcl-2 expression. Nevertheless, cyclin D1 knockdown did not induce resistance against venetoclax excluding a direct role for cyclin D1 in venetoclax sensitivity. The biology of this heterogeneity therefore remains unknown. Of interest, RNA-and *Assay for transposase-accessible chromatin (ATAC)*-sequencing recently indicated an association of remnants of B-cell biology with BCL2 dependency. These data therefore suggest the existence of biomarkers that could indicate venetoclax-sensitivity in MM, independent of t(11;14) [89]. Venetoclax in combination with Vd in MM patients with t(11;14) or high Bcl-2 expression (phase III **BELLINI trial,** NCT02755597: Ven-Vd vs. Vd) achieved an ORR of 84% vs. 70% for Ven-Vd with a median PFS of 23.2 vs. 11.4 months. In contrast, an increase in deaths was observed with venetoclax-Vd in the absence of t(11;14) and/or high Bcl2 levels, commonly due to infections and in the context of progressive disease. Based on these results, venetoclax is expected to be approved as the first precision medicine for MM therapy over the next months [90]. A phase II trial (NCT02899052) of venetoclax in combination with Kd showed promising response rates in RRMM patients, again with greater response rates in patients with t(11;14) [91]. Additional clinical trials evaluating venetoclax-containing combinations are ongoing. 

*Targeting epigenetic factors.* Targeting epigenetic factors has similarly become a promising therapeutic approach in MM. Importantly, due to its pleiotropic effects on intracellular pathways [92], the oral histone deacetylase (HDAC) inhibitor panobinostat is able to recapture responses in heavily pre-treated patients. Indeed, based on the phase III PANORAMA-1 trial intraveneous panobinostat in combination with bortezomib and dexamethasone was approved for the treatment of MM patients after at least two prior therapies including PIs and IMiDs on 19 March 2015 (phase III **PANORAMA-1 trial,** NCT01023308) [93]. Recent data of the phase II PANORAMA-3 trial demonstrated that the saftey profile of this regimen was improved upon replacing the previously used intravenous bortezomib with subcuteneous bortezomib [94]. Additional approaches to target epigenetic factors in MM include the use of EZH2-, HDAC6-and BET-inhibitors [85]. 

*Targeting MM driver mutations.* Given the increasing genomic complexity of the disease over time, some experts recommend early prevention strategies in patients with MGUS and SMM. Nevertheless, ongoing studies are also aiming at further dissecting inter-and intra-patient genomic heterogeneity patterns in RRMM cells in order to define distinct molecular subgroups, and to make them amendable to clinical decision-making and personalized targeted therapies. Importantly, only few genes are recurrently mutated (e.g., KRas/NRas, FAM46C, TP53, BRaf, TRAF3, and DIS3) and MM driver mutations are limited by vast intra-and inter-patient genetic heterogeneity. However, it must be noticed that mutational clusters exist, in the MAPK (~50%)-and the NFkB (~17%)-and PI3K/Akt (~17%) pathway, in particular. Nonetheless, even in the absence of these mutations, signaling pathways are activated through positive loops between the tumor cell and the cellular and non-cellular BM microenvironment. They result in the production and secretion of growth factors, cytokines and inflammatory mediators by both tumor and stroma cells, the expansion, recruitment and activation of suppressor cells (i.e., MDSCs, Tregs, plasmocytoid DCs), impaired T cell responses, an increased PD-1 and LAG-3 exhausted phenotype; as well as osteoclast activation; and endothelial cell proliferation (e.g., reviewed in [95,96]). **Pathway-directed therapies** and **kinase inhibitors** have therefore become a major focus in RRMM. Several comprehensive recent articles excellently review the functional basics of signaling pathways in general; and of MAPK-, Stat3-and NFkB-signaling, in particular; as well as the pathophysiologic role of protein kinases and emerging inhibitors in MM [97,98,99]. If present, driver mutations may serve as biomarkers for personalized therapies in MM. Mutations of the MAPK-pathway, in particular, are the most frequently observed pathway mutations in MM. Of note, they are more frequent in the RRMM than in the NDMM setting (72% vs. 43–53%) [100,101,102]. Specifically, K-and N-Ras mutations followed by the activation of BRaf, Dis3, and FAM46C were observed in ~21% and 19% of NDMM patients and may increase to up to 80% in RRMM patients, mostly affecting N-Ras (NRAS Q61 mutation) [103,104]. Similarly, the prevalence of the BRaf V600E mutation increases from 4% in NDMM to about 8% in RRMM patients [101]. Clinical activity of the Ras inhibitor tipifarnib and the multi-kinase Raf/VEGFR-2/c-Kit/PDGFR inhibitor sorafenib has been limited. However, other Ras inhibitors are in the clinical pipeline. In addition, targeting the *Germinal Center Kinase* (GCK/MAP4K2) in Ras-mutant MM may offer an exciting novel therapeutic approach [105]. MEK inhibition demonstrated a significantly prolonged survival in a novel, NRasQ61/Myc-transgenic mouse model of highly malignant MM. Based on their results the authors emphasize the strong rationale to develop MEK inhibition-based therapies for treating advanced/relapsed MM [106]. Promising, although short responses, have already been reported upon treatment with the BRaf V600E-inhibitor vemurafenib in RRMM patients [107,108,109]. Several ongoing clinical trials investigate the anti-MM activity of BRaf inhibitors (dabrafenib, encorafenib) alone or in combination with MEK inhibitors (trametinib, cobimetinib, selumetinib, binimetinib), i.e., the GMMG-BIRMA, the NCT03091257, the NCT03312530, and the NCT02407509 trial. Moreover, rationally derived **precision medicine umbrella and basket trials** investigate pathway-directed therapies alone or in combination with conventional or next-generation novel therapies including the **MATCH**, **TAPUR**, **CAPTUR**, and **MyDrug** trial. Results are eagerly awaited and will show whether precision medicine is able to counteract the steadily increasing heterogeneity and complexity of genetic abnormalities as well as multiclonality in RRMM. 

Besides shedding light on the genomic complexity of MM and its progress from precursor conditions MGUS and SMM, advances in single cell sequencing have identified **evolutionary clonal changes** occurring during RRMM. The complexity of MM genetics becomes even more challenging when considering the **spatial genomic heterogeneity** of the disease. Indeed, multi-region sequencing has identified a positive correlation between the size of a nodular plasma cell infiltrate and the presence of subclones [110]. Clonal evolution drives tumor progression, dissemination, and relapse in MM. At the time of diagnosis tumors harbor between three and seven detectable subclones that continuously evolve throughout the treatment (linear evolution), branch (branching evolution), show differential clonal response or stable clonality [104,111]. More diverse clones may progress faster. In addition, the impact of the tumor microenvironment on the evolution of clones needs to be considered. Of note, by utilizing an innovative DNA-barcode clone-tracking system on a MM PrEDiCT (progression through evolution and dissemination of clonal tumor cells) xenograft mouse model, Shen et al. recently demonstrated that only few clones that successfully adapt to the BM microenvironment enter the circulation and colonize distant BM sites. 28 genes were predicted to be master regulators of MM progression, including HMGA1 and PA2G4 [112]. While direct mutations of drug target genes are rare (i.e., CRBN mutations during lenalidomide maintenance), relapse during treatment is predominantly induced by the emergence of high-risk subclones carrying mutations of APOBEC, TP53 and IGLL5, Xbp-1, and MAPK [113]. For example, APOBEC activity is associated with increased mutational burden and shorter time to relapse. APOBEC family members have therefore been proposed as promising novel therapeutic targets (Figure 2).

#### 3.1.2. Assessment of MRD for Clinical Decision-Making and Personalized Targeted Therapies in RRMM

With high complete response rates observed with “novel” and “next generation novel” agents came the need for detecting deeper responses. The determination of MRD negativity by multicolor flow cytometry/next generation fluorescence (NGF) or next generation sequencing (NGS) in BM biopsies derived from MM patients represents a powerful prognostic marker utilized in various clinical trials. Nevertheless, its clinical utility as a tool to guide individualized patient-treatment strategies in ND but also RRMM patients is still under investigation. To get more insight, MRD status should be regularly evaluated over the course of the disease. In order to repetitively assess the MRD status in (RR)MM, tracking Circulating Tumor Cells (CTCs) or circulating free DNA (cfDNA) fragments from tumor cells in the peripheral blood (“liquid biopsies”) emerges as a promising, innovative, multi-regional minimally-invasive diagnostic method [114,115,116]. Indeed, cfDNA in the peripheral blood plasma of patients represents an informative biomarker for MM relapse [117]. Moreover, circulating micro-193a-5p may serve as a predictive marker of early relapse after autologous stem cell transplantation in MM patients [118]. Most recently, mass spectroscopy has been proposed as a complementary approach for the assessment of the MRD status in the peripheral blood [119,120]. Of note, a significant correlation of the MRD negativity determined by NGF in bone biopsies and by MALDI-TOF in the peripheral blood was reported at ASCO 2021 [121,122].

### 3.2. Innovative Approaches to Treat MM-Specific Vulnerabilities

#### 3.2.1. Novel Strategies to Target the UPR

Proteasome inhibitors target the MM-specific vulnerability of aberrant protein turnover and thereby normalize the imbalance of proteasome degradative capacity and proteasome load by inducing the terminal unfolded protein response (UPR), and subsequent cell death [123,124]. In addition to second-generation PIs carfilzomib, ixazomib, and marizomib, targeting deubiquitylating enzymes (DUBs), the 19S proteasome-associated ubiquitin receptor Rpn13, and the aggresome/autophagy pathway by HDAC6 inhibition may not only represent alternative approaches to target the UPR cascade, but may also to overcome bortezomib-resistance [125,126,127,128]. Besides direct tumor cell toxicity, these agents have numerous additional functions, BM angiogenesis and bone resorption in particular. Moreover, blocking the UPR cascade induces immunogenic cell death (ICD) (e.g., by bortezomib the type 1 IFN signature) *via* calreticulin expression (“eat-me-signal”) and the cGAS/STING pathway activation [129,130], whilst targeting Rpn13 in dendritic cells triggers T and NK cell anti-MM immunity [131].

#### 3.2.2. Protein Degradation: A Novel Approach to Target Tumor-Specific Molecules 

The recent discovery that IMiDs (thalidomide, lenalidomide, pomalidomide) co-opt and enhance activity of the E3-ubiquitin ligase CRBN complex and thereby selectively enhance ubiquitination and degradation of target proteins (i.e., the zinc finger TFs IKZ1/Ikaros and IKZ3/Aiolos) followed by a decrease of downstream cMyc and IRF-4 gave rise to the new therapeutic paradigm of protein degradation [132,133]. Based on these pivotal finding stronger chemoproteomic *CRBN E3 Ligase Modulator*/binder agents (“glues”; CELMoDs^®^) have been developed. Preclinical studies demonstrating that the CELMoD **iberdomide** (CC-220) has greater anti-MM activity than lenalidomide or pomalidomide in both IMiD-sensitive and -resistant MM cell lines provided the strong translational rationale for its development in combination with other agents for the treatment of RRMM [134]. Early results of clinical trials demonstrated promising efficacy of iberdomide in combination with dexamethasone; and bortezomib or carfilzomib, dexamethasone and daratumumab; respectively, in heavily pre-treated patients with RRMM [135]. A clinical trial evaluating iberdomide in combination with daratumumab and dexamethasone (Iberdomide-Dara-Dex) vs. Dara-Vd in RRMM patients will be initiated this fall (NCT04975997). **CC-92480**, another CELMoD, specifically designed for rapid protein degradation, achieved an ORR of up to 54.5% in a heavily pre-treated RRMM patient population (50% triple refractory) when used in combination with dexamethasone [136]. Several combination studies with CC-92480 are ongoing.

Moreover, the finding that IMiDs are co-opting CRBN, enhancing its activity and redirecting the protein degradation machinery of the cell toward the elimination of target proteins pioneered a new class of small-molecule medicines, *PROteolysis-TArgeting Chimeric molecules* (PROTACs) or degronomids. These novel agents hijack ubiquitin E3 ligases (besides CRBN, VHL and MDM2) for selective ubiquitination and degradation of disease-causing, target proteins (i.e., besides transcription factors such as IKZ1 and 3, c-Myc; also Brd4, Mcl-1, and USP7) [137]. Based on promising preclinical data, a clinical phase I/II trial testing the safety and tolerability of the single agent, orally bioavailable MonoDAC™ IZKF1/3 degrader CFT7455 is ongoing in RRMM (NCT04756726) [138] (Figure 2). 

## 4. Conclusions 

Based on translational research, a very exciting array of treatment options is currently available for RRMM patients. An immediate end to the constant expansion of our therapeutic armamentarium against (RR)MM is not foreseeable in the near future. Impressive response rates of 60 to 100% (compared to previously 30%) are obtained, especially with emerging immunotherapeutic agents (i.e., BiTEs, CAR T cells), even in heavily pre-treated patients. BCMA-targeting agents emerge as an additional backbone of RRMM therapy and will move to earlier lines of therapy in the near future. Decisions on the choice and sequencing of accessible therapeutic options have become increasingly complex but are required to ensure the best outcome for patients. Importantly, costs, accessibility, and QOL also need to be considered. We are confident that combination strategies of conventional, targeted, and immune therapies will continue to change rapidly, to further increase MRD negativity rates, to restore host anti-MM immunity, and to ultimately lead to long-term disease-free survival and potential cure of MM. 

## Figures and Tables

**Table 1:** Recent approvals for patients with relapsed/refractory multiple myeloma (RRMM).

FDA/EMA	Regimen	Study	Phase	Prior Lines	N	ORR, %	PFS, mos	OS	Ref.
FDA: 12 July 2021 EMA: 22 June 2021	Dara-Pd vs Pd	APOLLO	III	≥1 (Φ 2)	304	69 vs. 46	12.4 vs. 6.9 [HR: 0.69]	NR vs. NR	[15]
FDA: 31 March 2021 EMA: 19 April 2021	Isa-Kd vs Kd	IKEMA	III	1–3	302	86.6 vs. 82.9	NR vs. 19.15 [HR: 0.53]	NR vs. NR	[16]
FDA 21 December 2020	S-Vd	BOSTON	III	1–3 (Φ 2)	402	79 vs. 66	13.9 vs. 9.5 [HR: 0.70]	NR	[23]
20, 2020 EMA: 18 December 2020	Dara-Kd vs Kd	CANDOR	III	1–3	466	84.3 vs. 74.7	NR vs. 15.8 [HR: 0.59]	NR vs. NR	[6]
EMA: 16 May 2019	V-Pd vs Vd	OPTIMISSM	III	1–3 Φ 2	559	82.2 vs. 50 (ITT) 90 vs. 54.8 (after 1 prior tx)	HR (ITT): 0.61 [HR: after 1 prior line of Tx 0.54]	NR	[8]
FDA: 26 February 2021	Melflufen-Pd	HORIZON	II	≥2	157	31	5.7	20	[28]
FDA: 2 March 2020 EMA: 2 June 2020	Isa-Pd	ICARIA	III	≥2	307	60.4 vs. 35.3	12.7 vs. 7.9	1 yr OS: 72 vs. 63	[13]
FDA: 7 November 2020 EMA: 28 August 2019	Elo-Pd vs Pd	ELOQUENT-3	III	≥2 Φ 1–4	646	79 vs. 66	1 yr PFS 68 vs. 57 2 yrs PFS 41 vs. 27	NR	[10]
FDA: 5 August 2020 EMA: 26 August 2020	Belamaf	DREAMM-2	II	≥3 Φ 7	97 99	32 (2.5 mg/kg) 35 (3.4 mg/kg)	2.8 (2.5 mg/kg) 3.9 (3.4 mg/kg)	mOS estimate: 13.7 (2.5 mg/kg) 13.8 (3.4 mg/kg)	[34]
FDA: 27 March 2021 EMA: 19 August 2021	Ide-Cel	KarMMa	II	≥4 Φ 6	128	73	8.8 12.1 at 450 × 10^6^ 18 mos PFS 66%	19.4	[41]
FDA: 3 July 2019 EMA: 29 March 2021	Sd	STORM	I/II	Φ 7	79 122	26.2	3.7	8.6	[22]

Φ, median lines of prior therapy; mo/s, month/s; yr/s, year/s; PFS, progression free survival; OS, overall survival; HR, hazard ratio; NR, non-reached

## References

[1] Kumar S., Paiva B., Anderson K.C., Durie B., Landgren O., Moreau P., Munshi N., Lonial S., Bladé J., Mateos M.-V. (2016). International Myeloma Working Group Consensus Criteria for Response and Minimal Residual Disease Assessment in Multiple Myeloma. Lancet Oncol..

[2] Nooka A.K., Kastritis E., Dimopoulos M.A., Lonial S. (2015). Treatment Options for Relapsed and Refractory Multiple Myeloma. Blood.

[3] Rajkumar S.V., Harousseau J.-L., Durie B., Anderson K.C., Dimopoulos M., Kyle R., Blade J., Richardson P., Orlowski R., Siegel D. (2011). Consensus Recommendations for the Uniform Reporting of Clinical Trials: Report of the International Myeloma Workshop Consensus Panel 1. Blood.

[4] Moreau P., Kumar S.K., San Miguel J., Davies F., Zamagni E., Bahlis N., Ludwig H., Mikhael J., Terpos E., Schjesvold F. (2021). Treatment of Relapsed and Refractory Multiple Myeloma: Recommendations from the International Myeloma Working Group. Lancet Oncol..

[5] Bahlis N.J., Dimopoulos M.A., White D.J., Benboubker L., Cook G., Leiba M., Ho P.J., Kim K., Takezako N., Moreau P. (2020). Daratumumab plus Lenalidomide and Dexamethasone in Relapsed/Refractory Multiple Myeloma: Extended Follow-up of POLLUX, a Randomized, Open-Label, Phase 3 Study. Leukemia.

[6] Dimopoulos M., Quach H., Mateos M.-V., Landgren O., Leleu X., Siegel D., Weisel K., Yang H., Klippel Z., Zahlten-Kumeli A. (2020). Carfilzomib, Dexamethasone, and Daratumumab versus Carfilzomib and Dexamethasone for Patients with Relapsed or Refractory Multiple Myeloma (CANDOR): Results from a Randomised, Multicentre, Open-Label, Phase 3 Study. Lancet.

[7] Leleu X., Beksac M., Chou T., Dimopoulos M., Yoon S.-S., Prince H.M., Pour L., Shelekhova T., Chari A., Khurana M. (2021). Efficacy and Safety of Weekly Carfilzomib (70 Mg/M2), Dexamethasone, and Daratumumab (KdD70) Is Comparable to Twice-Weekly KdD56 While Being a More Convenient Dosing Option: A Cross-Study Comparison of the CANDOR and EQUULEUS Studies. Leuk. Lymphoma.

[8] Richardson P.G., Oriol A., Beksac M., Liberati A.M., Galli M., Schjesvold F., Lindsay J., Weisel K., White D., Facon T. (2019). Pomalidomide, Bortezomib, and Dexamethasone for Patients with Relapsed or Refractory Multiple Myeloma Previously Treated with Lenalidomide (OPTIMISMM): A Randomised, Open-Label, Phase 3 Trial. Lancet Oncol..

[9] Dimopoulos M., Weisel K., Moreau P., Anderson L.D., White D., San-Miguel J., Sonneveld P., Engelhardt M., Jenner M., Corso A. (2021). Pomalidomide, Bortezomib, and Dexamethasone for Multiple Myeloma Previously Treated with Lenalidomide (OPTIMISMM): Outcomes by Prior Treatment at First Relapse. Leukemia.

[10] Dimopoulos M.A., Dytfeld D., Grosicki S., Moreau P., Takezako N., Hori M., Leleu X., LeBlanc R., Suzuki K., Raab M.S. (2018). Elotuzumab plus Pomalidomide and Dexamethasone for Multiple Myeloma. N. Engl. J. Med..

[11] Dimopoulos M.A., Leleu X., Moreau P., Richardson P.G., Liberati A.M., Harrison S.J., Miles Prince H., Ocio E.M., Assadourian S., Campana F. (2020). Isatuximab plus Pomalidomide and Dexamethasone in Relapsed/Refractory Multiple Myeloma Patients with Renal Impairment: ICARIA-MM Subgroup Analysis. Leukemia.

[12] Chari A., Suvannasankha A., Fay J.W., Arnulf B., Kaufman J.L., Ifthikharuddin J.J., Weiss B.M., Krishnan A., Lentzsch S., Comenzo R. (2017). Daratumumab plus Pomalidomide and Dexamethasone in Relapsed and/or Refractory Multiple Myeloma. Blood.

[13] Attal M., Richardson P.G., Rajkumar S.V., San-Miguel J.J., Beksac M., Spicka I., Leleu X., Schjesvold F., Moreau P., Dimopoulos M.A. (2019). Isatuximab plus Pomalidomide and Low-Dose Dexamethasone versus Pomalidomide and Low-Dose Dexamethasone in Patients with Relapsed and Refractory Multiple Myeloma (ICARIA-MM): A Randomised, Multicentre, Open-Label, Phase 3 Study. Lancet.

[14] Richardson P.G., Perrot A.F., San-Miguel J., Beksac M., Spicka I., Leleu X., Schjesvold F., Moreau P., Dimopoulos M.A., Huang J.S. (2021). Updates from ICARIA-MM, a Phase 3 Study of Isatuximab (Isa) plus Pomalidomide and Low-Dose Dexamethasone (Pd) versus Pd in Relapsed and Refractory Multiple Myeloma (RRMM). J. Clin. Oncol..

[15] Dimopoulos M.A., Terpos E., Boccadoro M., Delimpasi S., Beksac M., Katodritou E., Moreau P., Baldini L., Symeonidis A., Bila J. (2021). Daratumumab plus Pomalidomide and Dexamethasone versus Pomalidomide and Dexamethasone Alone in Previously Treated Multiple Myeloma (APOLLO): An Open-Label, Randomised, Phase 3 Trial. Lancet Oncol..

[16] Moreau P., Dimopoulos M.-A., Mikhael J., Yong K., Capra M., Facon T., Hajek R., Špička I., Baker R., Kim K. (2021). Isatuximab, Carfilzomib, and Dexamethasone in Relapsed Multiple Myeloma (IKEMA): A Multicentre, Open-Label, Randomised Phase 3 Trial. Lancet.

[17] Spicka I., Moreau P., Martin T.G., Facon T., Martinez G., Oriol A., Koh Y., Lim A., Mikala G., Rosiñol L. (2021). Isatuximab plus Carfilzomib and Dexamethasone in Relapsed Multiple Myeloma Patients with High-Risk Cytogenetics: IKEMA Subgroup Analysis. J. Clin. Oncol..

[18] Facon T., Moreau P., Martin T.G., Spicka I., Oriol A., Koh Y., Lim A., Mikala G., Rosiñol L., Yağci M. (2021). Isatuximab plus Carfilzomib and Dexamethasone versus Carfilzomib and Dexamethasone in Elderly Patients with Relapsed Multiple Myeloma: IKEMA Subgroup Analysis. J. Clin. Oncol..

[19] Hajek R., Moreau P., Augustson B., Castro N., Pika T., Delimpasi S., de La Rubia J., Maiolino A., Reiman T.J., Kryuchkova I. (2021). Isatuximab plus Carfilzomib and Dexamethasone in Patients with Relapsed Multiple Myeloma According to Prior Lines of Treatment and Refractory Status: IKEMA Subgroup Analysis. J. Clin. Oncol..

[20] Costa L.J., Hari P., Kumar S.K., Tang S., Gandhi U.H., Shah J.J., Jagannath S., Chari A., Lakshman A., Shacham S. (2019). Overall Survival of Triple Class Refractory, Penta-Exposed Multiple Myeloma (MM) Patients Treated with Selinexor plus Dexamethasone or Conventional Care: A Combined Analysis of the STORM and Mammoth Studies. Blood.

[21] Podar K., Shah J., Chari A., Richardson P.G., Jagannath S. (2020). Selinexor for the Treatment of Multiple Myeloma. Expert Opin. Pharmacother..

[22] Chari A., Vogl D.T., Gavriatopoulou M., Nooka A.K., Yee A.J., Huff C.A., Moreau P., Dingli D., Cole C., Lonial S. (2019). Oral Selinexor-Dexamethasone for Triple-Class Refractory Multiple Myeloma. N. Engl. J. Med..

[23] Grosicki S., Simonova M., Spicka I., Pour L., Kriachok I., Gavriatopoulou M., Pylypenko H., Auner H.W., Leleu X., Doronin V. (2020). Once-per-Week Selinexor, Bortezomib, and Dexamethasone versus Twice-per-Week Bortezomib and Dexamethasone in Patients with Multiple Myeloma (BOSTON): A Randomised, Open-Label, Phase 3 Trial. Lancet.

[24] Bahlis N.J., Kotb R., Sebag M., Sutherland H.J., LeBlanc R., White D., Venner C.P., Kouroukis T., Bergstrom D., McCurdy A. (2016). Selinexor in Combination with Bortezomib and Dexamethasone (SdB) Demonstrates Significant Activity in Patients with Refractory Multiple Myeloma (MM) Including Proteasome-Inhibitor Refractory Patients: Results of the Phase I Stomp Trial. Blood.

[25] Gasparetto C., Lipe B., Tuchman S., Callander N.S., Lentzsch S., Baljevic M., Rossi A.C., Bahlis N.J., White D., Chen C. (2020). Once Weekly Selinexor, Carfilzomib, and Dexamethasone (SKd) in Patients with Relapsed/Refractory Multiple Myeloma (MM). J. Clin. Oncol..

[26] Chen C., Bahlis N., Gasparetto C., Tuchman S.A., Lipe B., Baljevic M., Kotb R., Sutherland H.J., Bensinger W.I., Sebag M. (2020). Selinexor in Combination with Pomalidomide and Dexamethasone (SPd) for Treatment of Patients with Relapsed Refractory Multiple Myeloma (RRMM). Blood.

[27] Gasparetto C., Lentzsch S., Schiller G.J., Callander N.S., Tuchman S., Bahlis N.J., White D., Chen C., Baljevic M., Sutherland H.J. (2020). Selinexor, Daratumumab, and Dexamethasone in Patients with Relapsed/Refractory Multiple Myeloma (MM). J. Clin. Oncol..

[28] Richardson P.G., Oriol A., Larocca A., Bladé J., Cavo M., Rodriguez-Otero P., Leleu X., Nadeem O., Hiemenz J.W., Hassoun H. (2021). Melflufen and Dexamethasone in Heavily Pretreated Relapsed and Refractory Multiple Myeloma. J. Clin. Oncol..

[29] Dhakal B., D’Souza A., Kleman A., Chhabra S., Mohan M., Hari P. (2021). Salvage Second Transplantation in Relapsed Multiple Myeloma. Leukemia.

[30] Garderet L., Iacobelli S., Koster L., Goldschmidt H., Johansson J.-E., Bourhis J.H., Krejci M., Leleu X., Potter M., Blaise D. (2018). Outcome of a Salvage Third Autologous Stem Cell Transplantation in Multiple Myeloma. Biol. Blood Marrow Transpl..

[31] Cook G., Ashcroft A.J., Cairns D.A., Williams C.D., Brown J.M., Cavenagh J.D., Snowden J.A., Parrish C., Yong K., Cavet J. (2016). The Effect of Salvage Autologous Stem-Cell Transplantation on Overall Survival in Patients with Relapsed Multiple Myeloma (Final Results from BSBMT/UKMF Myeloma X Relapse [Intensive]): A Randomised, Open-Label, Phase 3 Trial. Lancet Haematol..

[32] Cho S.-F., Lin L., Xing L., Li Y., Yu T., Anderson K.C., Tai Y.-T. (2020). BCMA-Targeting Therapy: Driving a New Era of Immunotherapy in Multiple Myeloma. Cancers.

[33] Sanchez L., Dardac A., Madduri D., Richard S., Richter J. (2021). B-Cell Maturation Antigen (BCMA) in Multiple Myeloma: The New Frontier of Targeted Therapies. Ther. Adv. Hematol..

[34] Lonial S., Lee H.C., Badros A., Trudel S., Nooka A.K., Chari A., Abdallah A.-O., Callander N., Lendvai N., Sborov D. (2020). Belantamab Mafodotin for Relapsed or Refractory Multiple Myeloma (DREAMM-2): A Two-Arm, Randomised, Open-Label, Phase 2 Study. Lancet Oncol..

[35] Bruins W.S.C., Zheng W., Higgins J.P., Willert E.K., Newcomb J., Dash A.B., Van De Donk N.W.C.J., Zweegman S., Mutis T. (2020). TAK-169, a Novel Recombinant Immunotoxin Specific for CD38, Induces Powerful Preclinical Activity against Patient-Derived Multiple Myeloma Cells. Blood.

[36] Vogl D.T., Kaufman J.L., Holstein S.A., Nadeem O., O’Donnell E., Suryanarayan K., Collins S., Parot X., Chaudhry M. (2020). TAK-573, an Anti-CD38/Attenuated Ifnα Fusion Protein, Has Clinical Activity and Modulates the Ifnα Receptor (IFNAR) Pathway in Patients with Relapsed/Refractory Multiple Myeloma. Blood.

[37] Figueroa-Vazquez V., Ko J., Breunig C., Baumann A., Giesen N., Pálfi A., Müller C., Lutz C., Hechler T., Kulke M. (2021). HDP-101, an Anti-BCMA Antibody-Drug Conjugate, Safely Delivers Amanitin to Induce Cell Death in Proliferating and Resting Multiple Myeloma Cells. Mol. Cancer Ther..

[38] Xing L., Wang S., Liu J., Yu T., Chen H., Wen K., Li Y., Lin L., Hsieh P.A., Cho S.-F. (2021). BCMA-Specific ADC MEDI2228 and Daratumumab Induce Synergistic Myeloma Cytotoxicity via IFN-Driven Immune Responses and Enhanced CD38 Expression. Clin. Cancer Res..

[39] Tai Y.-T., Mayes P.A., Acharya C., Zhong M.Y., Cea M., Cagnetta A., Craigen J., Yates J., Gliddon L., Fieles W. (2014). Novel Anti-B-Cell Maturation Antigen Antibody-Drug Conjugate (GSK2857916) Selectively Induces Killing of Multiple Myeloma. Blood.

[40] Lee H.C., Raje N.S., Landgren O., Upreti V.V., Wang J., Avilion A.A., Hu X., Rasmussen E., Ngarmchamnanrith G., Fujii H. (2021). Phase 1 Study of the Anti-BCMA Antibody-Drug Conjugate AMG 224 in Patients with Relapsed/Refractory Multiple Myeloma. Leukemia.

[41] Munshi N.C., Anderson L.D., Shah N., Madduri D., Berdeja J., Lonial S., Raje N., Lin Y., Siegel D., Oriol A. (2021). Idecabtagene Vicleucel in Relapsed and Refractory Multiple Myeloma. N. Engl. J. Med..

[42] Topp M.S., Duell J., Zugmaier G., Attal M., Moreau P., Langer C., Krönke J., Facon T., Salnikov A.V., Lesley R. (2020). Anti-B-Cell Maturation Antigen BiTE Molecule AMG 420 Induces Responses in Multiple Myeloma. J. Clin. Oncol..

[43] Harrison S.J., Minnema M.C., Lee H.C., Spencer A., Kapoor P., Madduri D., Larsen J., Ailawadhi S., Kaufman J.L., Raab M.S. (2020). A Phase 1 First in Human (FIH) Study of AMG 701, an Anti-B-Cell Maturation Antigen (BCMA) Half-Life Extended (HLE) BiTE (Bispecific T-Cell Engager) Molecule, in Relapsed/Refractory (RR) Multiple Myeloma (MM). Blood.

[44] Seckinger A., Delgado J.A., Moser S., Moreno L., Neuber B., Grab A., Lipp S., Merino J., Prosper F., Emde M. (2017). Target Expression, Generation, Preclinical Activity, and Pharmacokinetics of the BCMA-T Cell Bispecific Antibody EM801 for Multiple Myeloma Treatment. Cancer Cell.

[45] Costa L.J., Wong S.W., Bermúdez A., de la Rubia J., Mateos M.V., Ocio E.M., Rodríguez-Otero P., San Miguel J., Li S., Sarmiento R. Interim Results from the First Phase 1 Clinical Study of the B-Cell Maturation Antigen (BCMA) 2+1 T Cell Engager (TCE) CC-93269 in Patients with Relapsed/Refractory Multiple Myeloma. https://library.ehaweb.org/eha/2020/eha25th/295025/luciano.j.costa.interim.results.from.the.first.phase.1.clinical.study.of.the.

[46] Usmani S.Z., Garfall A.L., van de Donk N.W.C.J., Nahi H., San-Miguel J.F., Oriol A., Rosinol L., Chari A., Bhutani M., Karlin L. (2021). Teclistamab, a B-Cell Maturation Antigen × CD3 Bispecific Antibody, in Patients with Relapsed or Refractory Multiple Myeloma (MajesTEC-1): A Multicentre, Open-Label, Single-Arm, Phase 1 Study. Lancet.

[47] Madduri D., Rosko A., Brayer J., Zonder J., Bensinger W.I., Li J., Xu L., Adriaens L., Chokshi D., Zhang W. (2020). REGN5458, a BCMA × CD3 Bispecific Monoclonal Antibody, Induces Deep and Durable Responses in Patients with Relapsed/Refractory Multiple Myeloma (RRMM). Blood.

[48] Rodriguez C., D’Souza A., Shah N., Voorhees P.M., Buelow B., Vij R., Kumar S.K. (2020). Initial Results of a Phase I Study of TNB-383B, a BCMA × CD3 Bispecific T-Cell Redirecting Antibody, in Relapsed/Refractory Multiple Myeloma. Blood.

[49] Bahlis N.J., Raje N.S., Costello C., Dholaria B.R., Solh M.M., Levy M.Y., Tomasson M.H., Dube H., Liu F., Liao K.H. (2021). Efficacy and Safety of Elranatamab (PF-06863135), a B-Cell Maturation Antigen (BCMA)-CD3 Bispecific Antibody, in Patients with Relapsed or Refractory Multiple Myeloma (MM). J. Clin. Oncol..

[50] Berdeja J.G., Krishnan A.Y., Oriol A., van de Donk N.W.C.J., Rodríguez-Otero P., Askari E., Mateos M.-V., Minnema M.C., Costa L.J., Verona R. (2021). Updated Results of a Phase 1, First-in-Human Study of Talquetamab, a G Protein-Coupled Receptor Family C Group 5 Member D (GPRC5D) × CD3 Bispecific Antibody, in Relapsed/Refractory Multiple Myeloma (MM). J. Clin. Oncol..

[51] Sumiyoshi T., Nakamura R., Lear S., Wilson D., Choeurng V., Vaze A., Trudel S., Spencer A., Cohen A.D., Fonseca R. (2021). FCRH5 Target Expression in Patients with Relapsed Refractory Multiple Myeloma (RRMM) Treated with Cevostamab in an Ongoing Phase I Dose Escalation Study. EHA Libr..

[52] Bae J., Parayath N., Ma W., Amiji M., Munshi N., Anderson K.C. (2020). BCMA Peptide-Engineered Nanoparticles Enhance Induction and Function of Antigen-Specific CD8+ Cytotoxic T Lymphocytes against Multiple Myeloma: Clinical Applications. Leukemia.

[53] Martino M., Canale F.A., Alati C., Vincelli I.D., Moscato T., Porto G., Loteta B., Naso V., Mazza M., Nicolini F. (2021). CART-Cell Therapy: Recent Advances and New Evidence in Multiple Myeloma. Cancers.

[54] Mohyuddin G.R., Rooney A., Balmaceda N., Aziz M., Sborov D.W., McClune B., Kumar S.K. (2021). Chimeric Antigen Receptor T-Cell Therapy in Multiple Myeloma: A Systematic Review and Meta-Analysis of 950 Patients. Blood Adv..

[55] Raje N., Berdeja J., Lin Y., Siegel D., Jagannath S., Madduri D., Liedtke M., Rosenblatt J., Maus M.V., Turka A. (2019). Anti-BCMA CAR T-Cell Therapy Bb2121 in Relapsed or Refractory Multiple Myeloma. N. Engl. J. Med..

[56] Friedman K.M., Garrett T.E., Evans J.W., Horton H.M., Latimer H.J., Seidel S.L., Horvath C.J., Morgan R.A. (2018). Effective Targeting of Multiple B-Cell Maturation Antigen-Expressing Hematological Malignances by Anti-B-Cell Maturation Antigen Chimeric Antigen Receptor T Cells. Hum. Gene Ther..

[57] Fraietta J.A., Lacey S.F., Orlando E.J., Pruteanu-Malinici I., Gohil M., Lundh S., Boesteanu A.C., Wang Y., O’Connor R.S., Hwang W.-T. (2018). Determinants of Response and Resistance to CD19 Chimeric Antigen Receptor (CAR) T Cell Therapy of Chronic Lymphocytic Leukemia. Nat. Med..

[58] Berdeja J.G., Madduri D., Usmani S.Z., Jakubowiak A., Agha M., Cohen A.D., Stewart A.K., Hari P., Htut M., Lesokhin A. (2021). Ciltacabtagene Autoleucel, a B-Cell Maturation Antigen-Directed Chimeric Antigen Receptor T-Cell Therapy in Patients with Relapsed or Refractory Multiple Myeloma (CARTITUDE-1): A Phase 1b/2 Open-Label Study. Lancet.

[59] Agha M.E., Cohen A.D., Madduri D., Cohen Y.C., Delforge M., Hillengass J., Goldschmidt H., Weisel K., Raab M.-S., Scheid C. (2021). CARTITUDE-2: Efficacy and Safety of Ciltacabtagene Autoleucel (Cilta-Cel), a BCMA-Directed CAR T-Cell Therapy, in Patients with Progressive Multiple Myeloma (MM) after One to Three Prior Lines of Therapy. J. Clin. Oncol..

[60] Mailankody S., Jakubowiak A.J., Htut M., Costa L.J., Lee K., Ganguly S., Kaufman J.L., Siegel D.S.D., Bensinger W., Cota M. (2020). Orvacabtagene Autoleucel (Orva-Cel), a B-Cell Maturation Antigen (BCMA)-Directed CAR T Cell Therapy for Patients (Pts) with Relapsed/Refractory Multiple Myeloma (RRMM): Update of the Phase 1/2 EVOLVE Study (NCT03430011). J. Clin. Oncol..

[61] D’Agostino M., Raje N. (2020). Anti-BCMA CAR T-Cell Therapy in Multiple Myeloma: Can We Do Better?. Leukemia.

[62] Majzner R.G., Mackall C.L. (2019). Clinical Lessons Learned from the First Leg of the CAR T Cell Journey. Nat. Med..

[63] Samur M.K., Fulciniti M., Aktas Samur A., Bazarbachi A.H., Tai Y.-T., Prabhala R., Alonso A., Sperling A.S., Campbell T., Petrocca F. (2021). Biallelic Loss of BCMA as a Resistance Mechanism to CAR T Cell Therapy in a Patient with Multiple Myeloma. Nat. Commun..

[64] Da Vià M.C., Dietrich O., Truger M., Arampatzi P., Duell J., Heidemeier A., Zhou X., Danhof S., Kraus S., Chatterjee M. (2021). Homozygous BCMA Gene Deletion in Response to Anti-BCMA CAR T Cells in a Patient with Multiple Myeloma. Nat. Med..

[65] Frerichs K.A., Broekmans M.E.C., Marin Soto J.A., van Kessel B., Heymans M.W., Holthof L.C., Verkleij C.P.M., Boominathan R., Vaidya B., Sendecki J. (2020). Preclinical Activity of JNJ-7957, a Novel BCMA × CD3 Bispecific Antibody for the Treatment of Multiple Myeloma, Is Potentiated by Daratumumab. Clin. Cancer Res..

[66] Van de Donk N.W.C.J., Themeli M., Usmani S.Z. (2021). Determinants of Response and Mechanisms of Resistance of CAR T-Cell Therapy in Multiple Myeloma. Blood Cancer Discov..

[67] Yan Z., Cao J., Cheng H., Qiao J., Zhang H., Wang Y., Shi M., Lan J., Fei X., Jin L. (2019). A Combination of Humanised Anti-CD19 and Anti-BCMA CAR T Cells in Patients with Relapsed or Refractory Multiple Myeloma: A Single-Arm, Phase 2 Trial. Lancet Haematol..

[68] Li C., Mei H., Hu Y., Guo T., Liu L., Jiang H., Tang L., Wu Y., Ai L., Deng J. (2019). Improved Efficacy and Safety of a Dual-Target CAR-T Cell Therapy Targeting BCMA and CD38 for Relapsed/Refractory Multiple Myeloma from a Phase I Study. EHA Libr..

[69] Rodríguez-Lobato L.G., Ganzetti M., Fernández de Larrea C., Hudecek M., Einsele H., Danhof S. (2020). CAR T-Cells in Multiple Myeloma: State of the Art and Future Directions. Front. Oncol..

[70] Mailankody S., Matous J.V., Liedtke M., Sidana S., Malik S., Nath R., Oluwole O.O., Karski E.E., Lovelace W., Zhou X. (2020). Universal: An Allogeneic First-in-Human Study of the Anti-BCMA ALLO-715 and the Anti-CD52 ALLO-647 in Relapsed/Refractory Multiple Myeloma. Blood.

[71] Marofi F., Saleh M.M., Rahman H.S., Suksatan W., Al-Gazally M.E., Abdelbasset W.K., Thangavelu L., Yumashev A.V., Hassanzadeh A., Yazdanifar M. (2021). CAR-Engineered NK Cells; a Promising Therapeutic Option for Treatment of Hematological Malignancies. Stem Cell Res. Ther..

[72] Miller J.S., Rooney C.M., Curtsinger J., McElmurry R., McCullar V., Verneris M.R., Lapteva N., McKenna D., Wagner J.E., Blazar B.R. (2014). Expansion and Homing of Adoptively Transferred Human Natural Killer Cells in Immunodeficient Mice Varies with Product Preparation and in Vivo Cytokine Administration: Implications for Clinical Therapy. Biol. Blood Marrow Transpl..

[73] Lu H., Zhao X., Li Z., Hu Y., Wang H. (2021). From CAR-T Cells to CAR-NK Cells: A Developing Immunotherapy Method for Hematological Malignancies. Front. Oncol..

[74] Lin L., Cho S.-F., Xing L., Wen K., Li Y., Yu T., Hsieh P.A., Chen H., Kurtoglu M., Zhang Y. (2021). Preclinical Evaluation of CD8+ Anti-BCMA MRNA CAR T Cells for Treatment of Multiple Myeloma. Leukemia.

[75] Davis L.N., Sherbenou D.W. (2021). Emerging Therapeutic Strategies to Overcome Drug Resistance in Multiple Myeloma. Cancers.

[76] Ogiya D., Liu J., Ohguchi H., Kurata K., Samur M.K., Tai Y.-T., Adamia S., Ando K., Hideshima T., Anderson K.C. (2020). The JAK-STAT Pathway Regulates CD38 on Myeloma Cells in the Bone Marrow Microenvironment: Therapeutic Implications. Blood.

[77] Croft J., Ellis S., Sherborne A.L., Sharp K., Price A., Jenner M.W., Drayson M.T., Owen R.G., Chown S., Lindsay J. (2021). Copy Number Evolution and Its Relationship with Patient Outcome-an Analysis of 178 Matched Presentation-Relapse Tumor Pairs from the Myeloma XI Trial. Leukemia.

[78] Fonseca R., Debes-Marun C.S., Picken E.B., Dewald G.W., Bryant S.C., Winkler J.M., Blood E., Oken M.M., Santana-Dávila R., González-Paz N. (2003). The Recurrent IgH Translocations Are Highly Associated with Nonhyperdiploid Variant Multiple Myeloma. Blood.

[79] Smadja N.V., Bastard C., Brigaudeau C., Leroux D., Fruchart C., Groupe Français de Cytogénétique Hématologique (2001). Hypodiploidy Is a Major Prognostic Factor in Multiple Myeloma. Blood.

[80] Smadja N.V., Leroux D., Soulier J., Dumont S., Arnould C., Taviaux S., Taillemite J.L., Bastard C. (2003). Further Cytogenetic Characterization of Multiple Myeloma Confirms That 14q32 Translocations Are a Very Rare Event in Hyperdiploid Cases. Genes Chromosom. Cancer.

[81] Kumar S.K., Rajkumar S.V. (2018). The Multiple Myelomas—Current Concepts in Cytogenetic Classification and Therapy. Nat. Rev. Clin. Oncol..

[82] Manier S., Salem K.Z., Park J., Landau D.A., Getz G., Ghobrial I.M. (2017). Genomic Complexity of Multiple Myeloma and Its Clinical Implications. Nat. Rev. Clin. Oncol..

[83] Hoang P.H., Cornish A.J., Sherborne A.L., Chubb D., Kimber S., Jackson G., Morgan G.J., Cook G., Kinnersley B., Kaiser M. (2020). An Enhanced Genetic Model of Relapsed IGH-Translocated Multiple Myeloma Evolutionary Dynamics. Blood Cancer J..

[84] Walker B.A., Mavrommatis K., Wardell C.P., Ashby T.C., Bauer M., Davies F.E., Rosenthal A., Wang H., Qu P., Hoering A. (2018). Identification of Novel Mutational Drivers Reveals Oncogene Dependencies in Multiple Myeloma. Blood.

[85] Caprio C., Sacco A., Giustini V., Roccaro A.M. (2020). Epigenetic Aberrations in Multiple Myeloma. Cancers.

[86] Li S., Vallet S., Sacco A., Roccaro A., Lentzsch S., Podar K. (2019). Targeting Transcription Factors in Multiple Myeloma: Evolving Therapeutic Strategies. Expert Opin. Investig. Drugs.

[87] Chong P.S.Y., Chooi J.Y., Lim J.S.L., Toh S.H.M., Tan T.Z., Chng W.-J. (2021). SMARCA2 Is a Novel Interactor of NSD2 and Regulates Prometastatic PTP4A3 through Chromatin Remodeling in t(4;14) Multiple Myeloma. Cancer Res..

[88] Zhou J., Jia Y., Tan T.K., Chung T.-H., Sanda T., Chng W.J. (2019). Super-Enhancer Profiling Identifies Novel Oncogenes and Therapeutic Targets in Multiple Myeloma. Blood.

[89] Gupta V.A., Barwick B.G., Matulis S.M., Shirasaki R., Jaye D.L., Keats J.J., Oberlton B., Joseph N.S., Hofmeister C.C., Heffner L.T. (2021). Venetoclax Sensitivity in Multiple Myeloma Is Associated with B-Cell Gene Expression. Blood.

[90] Kumar S.K., Harrison S.J., Cavo M., de la Rubia J., Popat R., Gasparetto C., Hungria V., Salwender H., Suzuki K., Kim I. (2020). Venetoclax or Placebo in Combination with Bortezomib and Dexamethasone in Patients with Relapsed or Refractory Multiple Myeloma (BELLINI): A Randomised, Double-Blind, Multicentre, Phase 3 Trial. Lancet Oncol..

[91] Costa L.J., Davies F.E., Monohan G.P., Kovacsovics T.J., Burwick N., Jakubowiak A.J., Kaufman J.L., Hong W.-J., Dail M., Salem A.H. (2021). Phase 2 Study of Venetoclax plus Carfilzomib and Dexamethasone in Patients with Relapsed/Refractory Multiple Myeloma. Blood Adv..

[92] Hideshima T., Richardson P.G., Anderson K.C. (2011). Mechanism of Action of Proteasome Inhibitors and Deacetylase Inhibitors and the Biological Basis of Synergy in Multiple Myeloma. Mol. Cancer Ther..

[93] San-Miguel J.F., Hungria V.T.M., Yoon S.-S., Beksac M., Dimopoulos M.A., Elghandour A., Jedrzejczak W.W., Günther A., Nakorn T.N., Siritanaratkul N. (2016). Overall Survival of Patients with Relapsed Multiple Myeloma Treated with Panobinostat or Placebo plus Bortezomib and Dexamethasone (the PANORAMA 1 Trial): A Randomised, Placebo-Controlled, Phase 3 Trial. Lancet Haematol..

[94] Laubach J.P., Schjesvold F., Mariz M., Dimopoulos M.A., Lech-Maranda E., Spicka I., Hungria V.T., Shelekhova T., Abdo A., Jacobasch L. (2021). Efficacy and Safety of Oral Panobinostat plus Subcutaneous Bortezomib and Oral Dexamethasone in Patients with Relapsed or Relapsed and Refractory Multiple Myeloma (PANORAMA 3): An Open-Label, Randomised, Phase 2 Study. Lancet Oncol..

[95] Moschetta M., Kawano Y., Podar K. (2016). Targeting the Bone Marrow Microenvironment. Cancer Treat. Res..

[96] Lomas O.C., Tahri S., Ghobrial I.M. (2020). The Microenvironment in Myeloma. Curr. Opin. Oncol..

[97] Lind J., Czernilofsky F., Vallet S., Podar K. (2019). Emerging Protein Kinase Inhibitors for the Treatment of Multiple Myeloma. Expert Opin. Emerg. Drugs.

[98] Chong P.S.Y., Chng W.-J., de Mel S. (2019). STAT3: A Promising Therapeutic Target in Multiple Myeloma. Cancers.

[99] Wong A.H.-H., Shin E.M., Tergaonkar V., Chng W.-J. (2020). Targeting NF-ΚB Signaling for Multiple Myeloma. Cancers.

[100] Chng W.J., Gonzalez-Paz N., Price-Troska T., Jacobus S., Rajkumar S.V., Oken M.M., Kyle R.A., Henderson K.J., Van Wier S., Greipp P. (2008). Clinical and Biological Significance of RAS Mutations in Multiple Myeloma. Leukemia.

[101] Xu J., Pfarr N., Endris V., Mai E.K., Md Hanafiah N.H., Lehners N., Penzel R., Weichert W., Ho A.D., Schirmacher P. (2017). Molecular Signaling in Multiple Myeloma: Association of RAS/RAF Mutations and MEK/ERK Pathway Activation. Oncogenesis.

[102] Kortüm K.M., Mai E.K., Hanafiah N.H., Shi C.-X., Zhu Y.-X., Bruins L., Barrio S., Jedlowski P., Merz M., Xu J. (2016). Targeted Sequencing of Refractory Myeloma Reveals a High Incidence of Mutations in CRBN and Ras Pathway Genes. Blood.

[103] Morgan G.J., Walker B.A., Davies F.E. (2012). The Genetic Architecture of Multiple Myeloma. Nat. Rev. Cancer.

[104] Bolli N., Avet-Loiseau H., Wedge D.C., Van Loo P., Alexandrov L.B., Martincorena I., Dawson K.J., Iorio F., Nik-Zainal S., Bignell G.R. (2014). Heterogeneity of Genomic Evolution and Mutational Profiles in Multiple Myeloma. Nat. Commun..

[105] Li S., Fu J., Yang J., Ma H., Bhutani D., Mapara M.Y., Marcireau C., Lentzsch S. (2021). Targeting the GCK Pathway: A Novel and Selective Therapeutic Strategy against RAS-Mutated Multiple Myeloma. Blood.

[106] Wen Z., Rajagopalan A., Flietner E.D., Yun G., Chesi M., Furumo Q., Burns R.T., Papadas A., Ranheim E.A., Pagenkopf A.C. (2021). Expression of NrasQ61R and MYC Transgene in Germinal Center B Cells Induces a Highly Malignant Multiple Myeloma in Mice. Blood.

[107] Andrulis M., Lehners N., Capper D., Penzel R., Heining C., Huellein J., Zenz T., von Deimling A., Schirmacher P., Ho A.D. (2013). Targeting the BRAF V600E Mutation in Multiple Myeloma. Cancer Discov..

[108] Mey U.J.M., Renner C., von Moos R. (2017). Vemurafenib in Combination with Cobimetinib in Relapsed and Refractory Extramedullary Multiple Myeloma Harboring the BRAF V600E Mutation. Hematol. Oncol..

[109] Sharman J.P., Chmielecki J., Morosini D., Palmer G.A., Ross J.S., Stephens P.J., Stafl J., Miller V.A., Ali S.M. (2014). Vemurafenib Response in 2 Patients with Posttransplant Refractory BRAF V600E-Mutated Multiple Myeloma. Clin. Lymphoma Myeloma Leuk..

[110] Rasche L., Chavan S.S., Stephens O.W., Patel P.H., Tytarenko R., Ashby C., Bauer M., Stein C., Deshpande S., Wardell C. (2017). Spatial Genomic Heterogeneity in Multiple Myeloma Revealed by Multi-Region Sequencing. Nat. Commun..

[111] Szalat R., Avet-Loiseau H., Munshi N.C. (2016). Gene Expression Profiles in Myeloma: Ready for the Real World?. Clin. Cancer Res..

[112] Shen Y.J., Mishima Y., Shi J., Sklavenitis-Pistofidis R., Redd R.A., Moschetta M., Manier S., Roccaro A.M., Sacco A., Tai Y.-T. (2021). Progression Signature Underlies Clonal Evolution and Dissemination of Multiple Myeloma. Blood.

[113] Bustoros M., Sklavenitis-Pistofidis R., Park J., Redd R., Zhitomirsky B., Dunford A.J., Salem K., Tai Y.-T., Anand S., Mouhieddine T.H. (2020). Genomic Profiling of Smoldering Multiple Myeloma Identifies Patients at a High Risk of Disease Progression. J. Clin. Oncol..

[114] Huhn S., Weinhold N., Nickel J., Pritsch M., Hielscher T., Hummel M., Bertsch U., Huegle-Doerr B., Vogel M., Angermund R. (2017). Circulating Tumor Cells as a Biomarker for Response to Therapy in Multiple Myeloma Patients Treated within the GMMG-MM5 Trial. Bone Marrow Transpl..

[115] Mithraprabhu S., Khong T., Ramachandran M., Chow A., Klarica D., Mai L., Walsh S., Broemeling D., Marziali A., Wiggin M. (2017). Circulating Tumour DNA Analysis Demonstrates Spatial Mutational Heterogeneity That Coincides with Disease Relapse in Myeloma. Leukemia.

[116] Garcés J.-J., Bretones G., Burgos L., Valdes-Mas R., Puig N., Cedena M.-T., Alignani D., Rodriguez I., Puente D.Á., Álvarez M.-G. (2020). Circulating Tumor Cells for Comprehensive and Multiregional Non-Invasive Genetic Characterization of Multiple Myeloma. Leukemia.

[117] Yasui H., Kobayashi M., Sato K., Kondoh K., Ishida T., Kaito Y., Tamura H., Handa H., Tsukune Y., Sasaki M. (2021). Circulating Cell-Free DNA in the Peripheral Blood Plasma of Patients Is an Informative Biomarker for Multiple Myeloma Relapse. Int. J. Clin. Oncol..

[118] Park S.-S., Lim J.-Y., Kim T.W., Ko Y.H., Jeon W.-J., Lee S.-Y., Lee J.-H., Min C.-K. (2020). Predictive Impact of Circulating MicroRNA-193a-5p on Early Relapse after Autologous Stem Cell Transplantation in Patients with Multiple Myeloma. Br. J. Haematol..

[119] Derman B.A., Stefka A.T., Jiang K., McIver A., Kubicki T., Jasielec J.K., Jakubowiak A.J. (2021). Measurable Residual Disease Assessed by Mass Spectrometry in Peripheral Blood in Multiple Myeloma in a Phase II Trial of Carfilzomib, Lenalidomide, Dexamethasone and Autologous Stem Cell Transplantation. Blood Cancer J..

[120] Eveillard M., Rustad E., Roshal M., Zhang Y., Ciardiello A., Korde N., Hultcrantz M., Lu S., Shah U., Hassoun H. (2020). Comparison of MALDI-TOF Mass Spectrometry Analysis of Peripheral Blood and Bone Marrow-Based Flow Cytometry for Tracking Measurable Residual Disease in Patients with Multiple Myeloma. Br. J. Haematol..

[121] Dispenzieri A., Krishnan A.Y., Arendt B., Dasari S., Efebera Y.A., Geller N., Giralt S., Hahn T., Kohlhagen M.C., Landau H.J. (2021). MASS-FIX versus Standard Methods to Predict for PFS and OS among Multiple Myeloma Patients Participating on the STAMINA Trial. J. Clin. Oncol..

[122] Puig N., Mateos M.-V., Contreras T., Paiva B., Cedena M.T., Pérez J.J., Aires I., Agullo C., Martinez-Lopez J., Otero P.R. (2019). Qip-Mass Spectrometry in High Risk Smoldering Multiple Myeloma Patients Included in the GEM-CESAR Trial: Comparison with Conventional and Minimal Residual Disease IMWG Response Assessment. Blood.

[123] Cenci S., Mezghrani A., Cascio P., Bianchi G., Cerruti F., Fra A., Lelouard H., Masciarelli S., Mattioli L., Oliva L. (2006). Progressively Impaired Proteasomal Capacity during Terminal Plasma Cell Differentiation. EMBO J..

[124] Obeng E.A., Carlson L.M., Gutman D.M., Harrington W.J., Lee K.P., Boise L.H. (2006). Proteasome Inhibitors Induce a Terminal Unfolded Protein Response in Multiple Myeloma Cells. Blood.

[125] Song Y., Ray A., Li S., Das D.S., Tai Y.T., Carrasco R.D., Chauhan D., Anderson K.C. (2016). Targeting Proteasome Ubiquitin Receptor Rpn13 in Multiple Myeloma. Leukemia.

[126] Song Y., Park P.M.C., Wu L., Ray A., Picaud S., Li D., Wimalasena V.K., Du T., Filippakopoulos P., Anderson K.C. (2019). Development and Preclinical Validation of a Novel Covalent Ubiquitin Receptor Rpn13 Degrader in Multiple Myeloma. Leukemia.

[127] Chauhan D., Tian Z., Nicholson B., Kumar K.G.S., Zhou B., Carrasco R., McDermott J.L., Leach C.A., Fulcinniti M., Kodrasov M.P. (2012). A Small Molecule Inhibitor of Ubiquitin-Specific Protease-7 Induces Apoptosis in Multiple Myeloma Cells and Overcomes Bortezomib Resistance. Cancer Cell.

[128] Tian Z., D’Arcy P., Wang X., Ray A., Tai Y.-T., Hu Y., Carrasco R.D., Richardson P., Linder S., Chauhan D. (2014). A Novel Small Molecule Inhibitor of Deubiquitylating Enzyme USP14 and UCHL5 Induces Apoptosis in Multiple Myeloma and Overcomes Bortezomib Resistance. Blood.

[129] Galluzzi L., Buqué A., Kepp O., Zitvogel L., Kroemer G. (2017). Immunogenic Cell Death in Cancer and Infectious Disease. Nat. Rev. Immunol..

[130] Gulla A., Morelli E., Samur M.K., Botta C., Hideshima T., Bianchi G., Fulciniti M., Malvestiti S., Prabhala R.H., Talluri S. (2021). Bortezomib Induces Anti–Multiple Myeloma Immune Response Mediated by CGAS/STING Pathway Activation. Blood Cancer Discov..

[131] Ray A., Song Y., Chauhan D., Anderson K.C. (2019). Blockade of Ubiquitin Receptor Rpn13 in Plasmacytoid Dendritic Cells Triggers Anti-Myeloma Immunity. Blood Cancer J..

[132] Kronke J., Udeshi N.D., Narla A., Grauman P., Hurst S.N., McConkey M., Svinkina T., Heckl D., Comer E., Li X. (2014). Lenalidomide Causes Selective Degradation of IKZF1 and IKZF3 in Multiple Myeloma Cells. Science.

[133] Lu G., Middleton R.E., Sun H., Naniong M., Ott C.J., Mitsiades C.S., Wong K.-K., Bradner J.E., Kaelin W.G. (2014). The Myeloma Drug Lenalidomide Promotes the Cereblon-Dependent Destruction of Ikaros Proteins. Science.

[134] Bjorklund C.C., Kang J., Amatangelo M., Polonskaia A., Katz M., Chiu H., Couto S., Wang M., Ren Y., Ortiz M. (2020). Iberdomide (CC-220) Is a Potent Cereblon E3 Ligase Modulator with Antitumor and Immunostimulatory Activities in Lenalidomide- and Pomalidomide-Resistant Multiple Myeloma Cells with Dysregulated CRBN. Leukemia.

[135] Lonial S., Richardson P.G., Popat R., Stadtmauer E., Larsen J., Oriol A., Knop S., Jagannath S., Cook G., Badros A.Z. (2021). Iberdomide (Iber) in Combination with Dexamethasone (Des) and Daratumumab (Dara), Bortezomib (Boot), or Carfilzomib (Cfz) in Patients (Its) with Relapsed/Refractory Multiple Myeloma (RRMM). EHA Libr..

[136] Richardson P.G., Vangsted A.J., Ramasamy K., Trudel S., Martínez J., Mateos M.-V., Rodríguez Otero P., Lonial S., Popat R., Oriol A. (2020). First-in-Human Phase I Study of the Novel CELMoD Agent CC-92480 Combined with Dexamethasone (DEX) in Patients (Pts) with Relapsed/Refractory Multiple Myeloma (RRMM). J. Clin. Oncol..

[137] Winter G.E., Buckley D.L., Paulk J., Roberts J.M., Souza A., Dhe-Paganon S., Bradner J.E. (2015). Phthalimide Conjugation as a Strategy for in Vivo Target Protein Degradation. Science.

[138] Henderson J.A., Kirby R.J., Perino S., Agafonov R.V., Chaturvedi P., Class B., Cocozziello D., Eron S.J., Good A., Hart A.A. Abstract LB007: CFT7455: A Novel, IKZF1/3 Degrader That Demonstrates Potent Tumor Regression in IMiD-Resistant Multiple Myeloma (MM) Xenograft Models. Proceedings of the AACR (American Association for Cancer Research) Annual Meeting 2021.

